# Comparative Analysis of Toxic Responses of Organic Extracts from Diesel and Selected Alternative Fuels Engine Emissions in Human Lung BEAS-2B Cells

**DOI:** 10.3390/ijms17111833

**Published:** 2016-11-03

**Authors:** Helena Libalova, Pavel Rossner,, Kristyna Vrbova, Tana Brzicova, Jitka Sikorova, Michal Vojtisek-Lom, Vit Beranek, Jiri Klema, Miroslav Ciganek, Jiri Neca, Katerina Pencikova, Miroslav Machala, Jan Topinka

**Affiliations:** 1 Department of Genetic Toxicology and Nanotoxicology, Institute of Experimental Medicine AS CR, Videnska 1083, 142 20 Prague, Czech Republic; libalova@biomed.cas.cz (H.L.); prossner@biomed.cas.cz (P.R.Jr.); kristyna.vrbova@biomed.cas.cz (K.V.); tana.brzicova@biomed.cas.cz (T.B.); jitkastolcpartova@seznam.cz (J.S.); 2 Faculty of Safety Engineering, VSB–Technical University of Ostrava, Lumirova 13, 700 30 Ostrava, Czech Republic; 3 Institute for Environmental Studies, Faculty of Science, Charles University in Prague, Benatska 2, 128 01 Prague 2, Czech Republic; 4 Center of Vehicles for Sustainable Mobility, Faculty of Mechanical Engineering, Czech Technical University in Prague, Technicka 4, 166 07 Prague, Czech Republic; michal.vojtisek@fs.cvut.cz (M.V.-L.); vit.beranek@fs.cvut.cz (V.B.); 5 Department of Cybernetics, Faculty of Electrical Engineering, Czech Technical University in Prague, Karlovo namesti 13, 121 35 Prague, Czech Republic; klema@fel.cvut.cz; 6 Department of Chemistry and Toxicology, Veterinary Research Institute, Hudcova 296/70, 621 00 Brno, Czech Republic; ciganek@vri.cz (M.C.); neca@vri.cz (J.N.); pencikova@vri.cz (K.P.); machala@vri.cz (M.M.)

**Keywords:** diesel, alternative fuels, diesel exhaust particles, organic extracts, gene expression profiles

## Abstract

This study used toxicogenomics to identify the complex biological response of human lung BEAS-2B cells treated with organic components of particulate matter in the exhaust of a diesel engine. First, we characterized particles from standard diesel (B0), biodiesel (methylesters of rapeseed oil) in its neat form (B100) and 30% by volume blend with diesel fuel (B30), and neat hydrotreated vegetable oil (NEXBTL100). The concentration of polycyclic aromatic hydrocarbons (PAHs) and their derivatives in organic extracts was the lowest for NEXBTL100 and higher for biodiesel. We further analyzed global gene expression changes in BEAS-2B cells following 4 h and 24 h treatment with extracts. The concentrations of 50 µg extract/mL induced a similar molecular response. The common processes induced after 4 h treatment included antioxidant defense, metabolism of xenobiotics and lipids, suppression of pro-apoptotic stimuli, or induction of plasminogen activating cascade; 24 h treatment affected fewer processes, particularly those involved in detoxification of xenobiotics, including PAHs. The majority of distinctively deregulated genes detected after both 4 h and 24 h treatment were induced by NEXBTL100; the deregulated genes included, e.g., those involved in antioxidant defense and cell cycle regulation and proliferation. B100 extract, with the highest PAH concentrations, additionally affected several cell cycle regulatory genes and p38 signaling.

## 1. Introduction

Diesel engines are popular not only for trucks but also in passenger cars and a wide range of mobile machinery due to lower fuel consumption and consequently lower CO_2_ emissions, relatively low production and operating costs, reliability, and overall practicality. Awareness of global climate change and the need to reduce CO_2_ emissions have led to renewable diesel fuels with significantly lower emissions of fossil carbon (as CO_2_) compared to conventional fossil diesel fuels. Drop-in replacement fuels for existing diesel engines are typically produced from vegetable oils and other fatty acids, either by transesterification into n-alkyl-esters of fatty acids, called biodiesel (in the EU, typically rapeseed oil methylesters), or by thermal hydrodeoxynation or similar processes into mostly paraffinic fuels, termed hydrotreated vegetable oil (HVO). The chemical composition and properties of renewable diesels are dependent on the production technique; e.g., biodiesel is enriched with oxygen in comparison with HVO and conventional diesel, biodiesel and HVO lack sulfur and do not contain aromatic hydrocarbons as diesel [1,2].

Renewable diesel fuels produce less particulate matter, carbon monoxide and total hydrocarbons due to reduced aromatics content, higher cetane number (HVO), or oxygen content (biodiesel); the same property, however, could lead to higher emissions of nitrogen oxides (NO_x_) [3,4,5,6,7,8]. A disadvantage specific for biodiesel is its lower energy density, leading to a higher fuel consumption and a decrease in torque and power at full load conditions [9]. HVO, however, has no or minor negative influence on fuel consumption and engine power [3,10]. Particle number size distributions were mostly shifting toward smaller sizes with an increased portion of biodiesel in the fuel [4,11]. All presented emission patterns for biodiesel strongly depend on operating regime and engine type, therefore there are also opposite findings, e.g., an increase in particulate mass during idling on biodiesel [12] or opposite results in particle number concentrations in exhaust between two engines under the same conditions on the same biodiesel/diesel mixtures [13].

Diesel exhaust, along with air pollution, was categorized by the International Agency for Research on Cancer (IARC) as carcinogenic to humans [14,15]. However, the different chemical composition of diverse blends of diesel and renewable fuels also influences exhaust emission characteristics. In recent years, more attention has been paid to diesel exhaust particles (DEP) (reviewed in [16]), as DEP is a carrier of polycyclic aromatic hydrocarbons (PAHs) that exert most of the genotoxic activity. Benzo[a]pyrene (BaP) is carcinogenic to humans and several other PAHs have been classified as probable or possible human carcinogens [14]. Although secondary genotoxicity elicited by the interaction of the particles with the immune system resulting in inflammation is believed to be an important factor in the negative health effects of DEP exposure [17], primary genotoxicity directly caused by organic compounds adsorbed onto DEP may substantially contribute to the carcinogenicity of DEP. As the content of PAHs between different fuels’ DEPs varies, the genotoxicity of their organic extracts also depends on the fuel used [12,18]. It has been reported that toxic effects of DEP and DEP extracts from identical fuels differ [19,20]. Therefore, ideally, a battery of tests including both particles and extracts should be applied. However, to specifically reveal the potential carcinogenic effects of PAHs, application of organic extracts is more relevant. Although the genotoxicity of DEP extracts may be easily tested by standardized, well-established tests including cytotoxicity measurement [20], Ames test [20,21], analysis of double strand DNA breaks [21], apoptosis, frequency of micronuclei [22], bulky DNA adducts formation or oxidative DNA damage [12], for a comprehensive assessment of the biological processes induced by the DEP extracts the treatment of the whole genome gene expression analysis is a valuable tool.

The objective of the present study was to compare the toxicity of organic compound mixtures extracted from DEPs produced by diverse fuels (conventional fossil diesel, 100% biodiesel, a blend of diesel/biodiesel and HVO) with the special focus on characterization of the complex cellular response. A fuel marketed as “renewable diesel” under the name of NEXBTL (Neste Oil, Espoo, Finland) was used as a HVO representative. An IVECO Tector 5.9 L, 176 kW engine was used to generate DEPs as it is commonly used in the EU in smaller trucks. We aimed to investigate the whole-genome gene expression changes following the exposure to organic compounds extracted from different diesel/renewable diesel fuel exhaust particles and to reveal common genes and processes similarly affected upon all DEP extracts treatment as well as the specific molecular signatures linked to the activity of individual extracts. A detailed chemical analysis of PAHs and their derivatives was employed to clarify the differences in toxicity of individual extracts. To support gene expression data, the oxidative potential of DEP extracts was evaluated by intracellular reactive oxygen species (ROS) assay and glutathione assay upon 4 h exposure and expressions of selected genes were confirmed by RT-qPCR.

## 2. Results

### 2.1. Characteristics of Collected Exhaust Particles; Chemical Analysis of Organic Extracts

**Figure 1:**
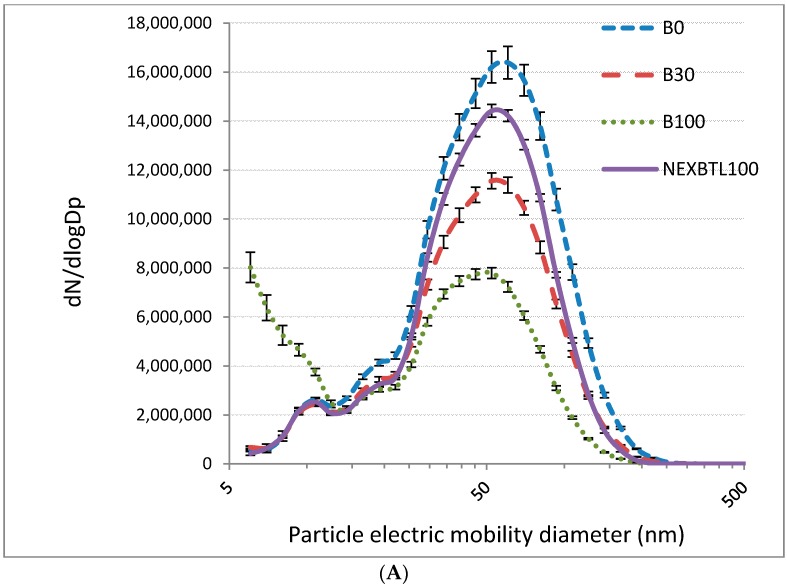

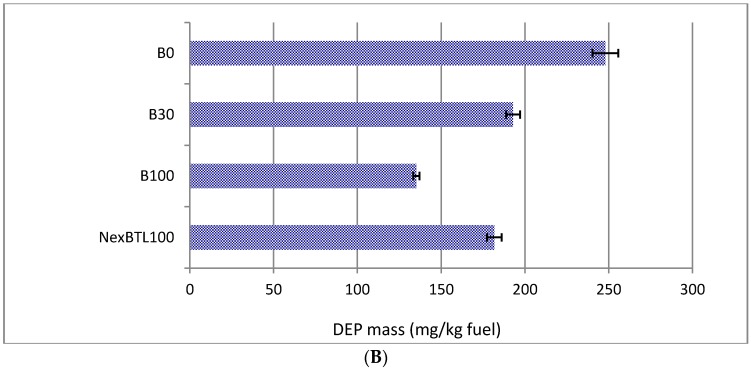
Characterization of collected particles: (**A**) number of particles per second as a function of their size (nm) for undiluted emissions from the tested fuels; and (**B**) the amount of particles (mg) per kg of individual fuels (average mass collected over 10 runs of WHTC cycle, see Materials and Methods). DEP, diesel exhaust particles.

Figure 1A shows particle number distributions for individual fuels. Although the size of most of the DEPs for all fuels was between 25 and 100 nm, B100 also generated a substantial amount of particles smaller than 10 nm. The total mass of particles emitted, calculated per kg of fuel, differed among diesel, biodiesel and NEXBTL100 (Figure 1B). While standard diesel fuel generated more than 11 mg particles/kg, for B100 the production was almost halved (6 mg/kg). NEXBTL100 and B30 fuels produced similar amount of particles (8.2 and 8.7 mg/kg, respectively). Next, we determined the content of PAHs and their derivatives in dichloromethane extracts of DEPs from individual fuels. In general, the content of PAHs was the lowest in the NEXBTL100 extract and highest in the extracts from DEPs from fuels containing biodiesel (B30 or B100) (Table 1). The sum of PAH concentrations classified by IARC as carcinogenic, or probably/possibly carcinogenic to humans (c-PAHs; Group 1, 2A or 2B) including benzo[a]pyrene, benz[a]anthracene, chrysene, benzo[b]fluoranthene, benzo[k]fluoranthene, dibenz[a,h]anthracene, and indeno[1,2,3-cd]pyrene, expressed in ng of cPAH per mg of particulate matter, ranged from 7.5 ng/mg for NEXBTL100 to 51.7 ng/mg for B100. Interestingly, although concentrations of most of the c-PAHs were lowest in the NEXBTL100 extract, this was not confirmed for BaP, a known human carcinogen. On the other hand, high concentrations of low-molecular-weight PAHs fluoranthene and pyrene were found in the B30 and B100 extracts (Table 1).

### 2.2. Cytotoxicity Assessment

**Figure 2:**
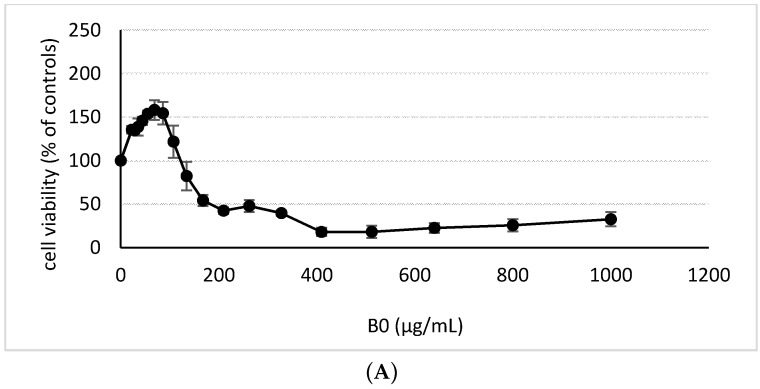

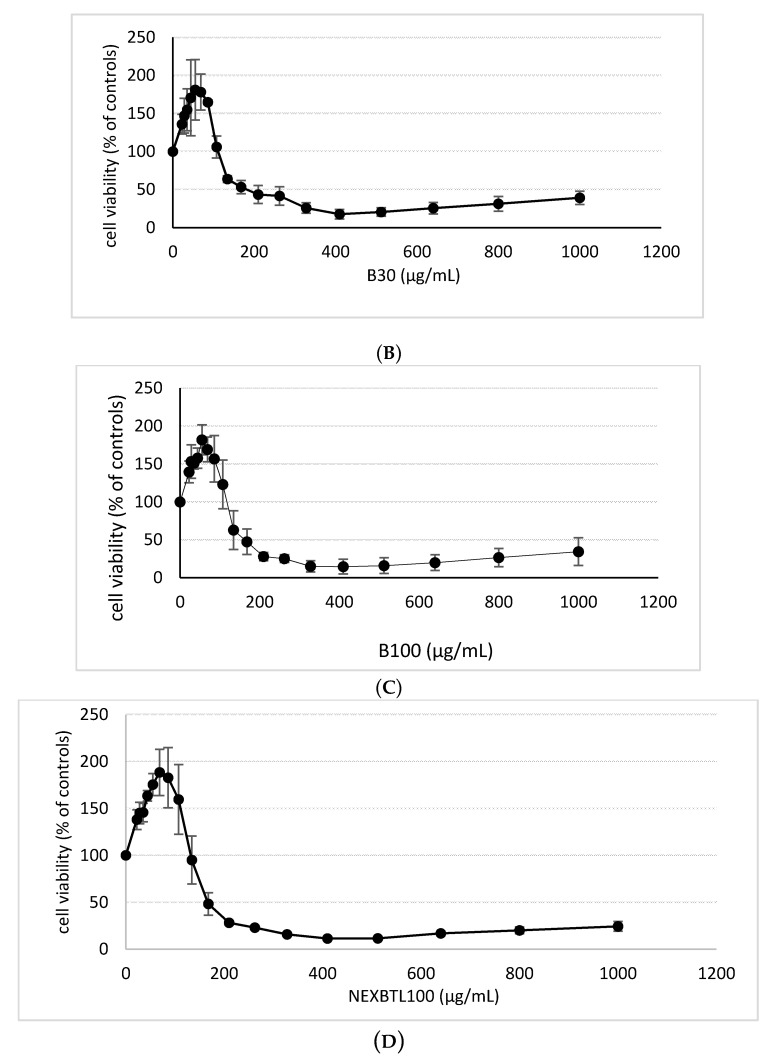
Cell viability evaluated by WST-1 Proliferation Assay. Cells were exposed to 18 different concentrations of: (**A**) B0; (**B**) B30; (**C**) B100; and (**D**) NEXBTL100 extracts for 24 h and the results were expressed as a percentage of increased/decreased activity of mitochondrial dehydrogenases to metabolize tetrazolium dye compared to the untreated control.

Cytotoxicity was evaluated upon 24 h exposure using the WST-1 Proliferation Assay. The range of concentrations from 22 μg/mL to 1000 μg/mL of each DEP extract was applied to obtain a dose–response proliferation capacity of the cells. The results for individual extracts were expressed as a percentage of increase/decrease of the potency to convert tetrazolium dye by mitochondrial dehydrogenase enzymes compared to the untreated control (Figure 2). We detected a substantial increase of absorbance up to the dose 68.7 μg/mL (154.5%–182.7%) while higher doses gradually decreased the absorbance upon all DEP extract treatments. Dose 134.2 μg/mL already caused inhibition of metabolic activity indicating decreased cell viability below the control level (95%–62.8%). NEXBTL100 extract exhibited the highest but not significant rate of dye conversion up to the dose 134.2 μg/mL compared to other treatments, however, the rapid decrease at higher doses was consequently observed. For all DEP extracts, a non-toxic concentration of 50 μg/mL was selected for further testing.

### 2.3. Quantification of Intracellularly Generated Reactive Oxygen Species and Reduced Glutathione Level

**Figure 3:**
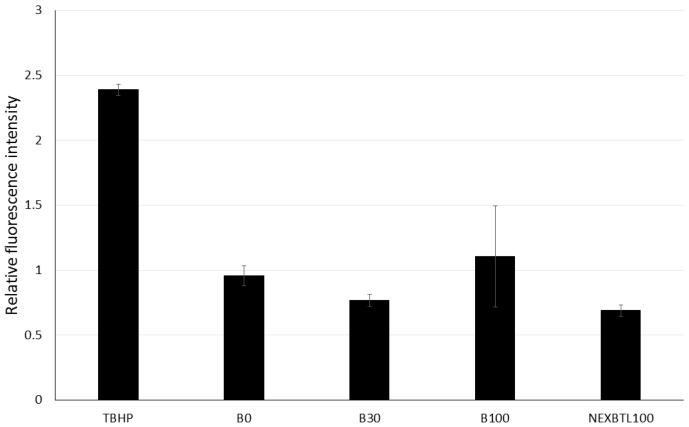
Relative intracellular ROS production upon 4 h exposure to DEP extract. Results are expressed as ratios of fluorescence intensity of treated and untreated cells. Cells were incubated with 50 μg/mL of different DEP extracts and 250 μM *tert*-butyl hydroperoxide (TBHP) as a positive control. No significant changes between the samples treated with individual DEP extracts and the control sample were found.

**Figure 4:**
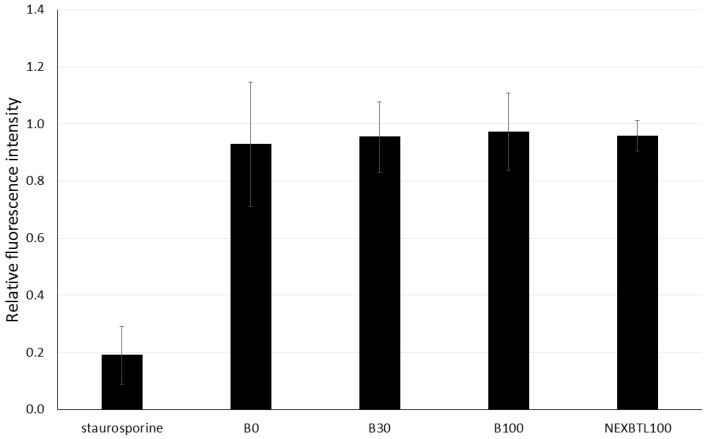
Relative GSH levels upon 4 h exposure to DEP extracts. Results are expressed as ratios of fluorescence intensity of treated and untreated cells. Cells were incubated with 50 μg/mL of different DEP extracts and staurosporine (1 μg/mL) as a positive control. No significant changes between the samples treated with individual DEP extracts and the control sample were found.

We measured the oxidative potency of DEP extracts, intracellular ROS production and levels of reduced glutathione in BEAS-2B cells. After 4 h incubation with 50 μg/mL dose of each DEP extract, we did not observe any significant increase of fluorescence indicating ROS production. Instead, NEXBTL100 and B30 rather slightly decreased (31% and 23%, respectively) ROS levels compared to untreated controls. B100 exhibited a subtle increase (11%) and B0 had no effect (Figure 3). Similar to ROS production, we did not find significant changes in reduced glutathione (GSH) levels among DEP-treated cells (Figure 4).

### 2.4. Gene Expression Profiling in BEAS-2B Exposed to Diverse DEP Extracts

#### 2.4.1. Identification of Differentially Expressed Genes

**Figure 5:**
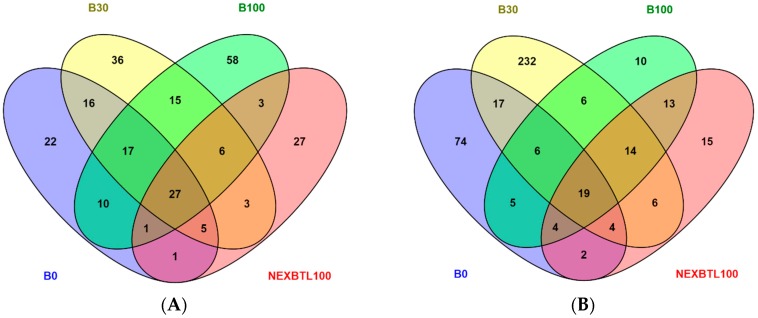
Venn diagrams illustrating the overlap of genes commonly modulated in response to all DEP extract treatments as well as numbers of specifically modulated genes by each individual DEP extract upon: (**A**) 4 h; and (**B**) 24 h incubation.

We performed differential gene expression analysis to explore the impact of DEP extract exposure on mRNA expression levels. Results showed that all DEP extracts induced significant changes in expression of various genes following both 4 and 24 h exposure. The number of upregulated and downregulated genes varied among all samples. After 4 h exposure, B100 extract elicited the most pronounced response and modulated the highest number of genes, while NEXBTL100 exhibited the lowest potency to induce gene expression changes. Interestingly, the number of deregulated genes increased with the increasing ratio of biocomponent in the fuel. After 24 h exposure, B30 extract yielded an increase of considerably higher number of deregulated genes compared to other treatments. Similar to 4 h treatment, 24 h exposure to NEXBTL100 resulted in the weakest response in terms of gene numbers. Venn diagrams were generated to visualize the common genes identically modulated by all DEP extracts as well as unique transcripts affected by individual treatments. The analysis revealed 27 common genes deregulated following 4 h exposure (Figure 5A). Several genes were common for other combinations of DEP extract treatments while each individual DEP extract induced a relatively higher number of specifically deregulated genes (22 for B0, 36 for B30, 58 for B100 extract treatment and 27 genes for NEXBTL100). Twenty-four hour exposure resulted in deregulation of 19 genes common for all treatments (Figure 5B). A substantially higher number of genes was modulated in response to individual DEP extracts, particularly B30 induced changes in expression of 232 genes compared to other extracts: NEXBTL100 extract induced deregulation of 15 genes; B0, 74 genes; and B100, 10 genes. A list of differentially deregulated genes is included in Table S1.

#### 2.4.2. Functional Analysis

##### Commonly Modulated Pathways

We performed functional analysis of significantly deregulated genes using the ToppFun tool to discover common processes and pathways identically modified by all DEP extract treatments. Table 2 shows the rank of pathways enriched in the list of 27 common genes deregulated after 4 h exposure. The top represented pathway was “Benzo[a]pyrene metabolism” with aldo-keto reductases (*AKRs*) *AKR1C3* and *AKR1C2* as the most important contributing genes. It was the only pathway with significance below 0.05 after Bonferroni correction. However, numerous other pathways were found with high significance after False Discovery Rate Benjamini–Hochberg (FDR B&H) correction. *AKR1C3* and *AKR1C2* were further involved in “Synthesis of bile acids and bile salts via 27-hydroxycholesterol”, “Synthesis of bile acids and bile salts via 24-hydroxycholesterol”, “Synthesis of bile acids and bile salts via 7α-hydroxycholesterol”, “Synthesis of bile acids and bile salts”, “Bile acid and bile salt metabolism” and together with thioredoxin reductase (*TXNRD1*), glioma pathogenesis-related protein 1 (*GLIPR1*), connective tissue growth factor (*CTGF*), low density lipoprotein receptor (*LDLR*) in “Metabolism of lipids and lipoproteins”. A combination of genes *TXNRD1*, *GLIPR1* and *CTGF* also played a role in “Regulation of Lipid Metabolism by Peroxisome proliferator-activated receptor alpha (PPARalpha)” and “PPARA Activates Gene Expression” pathways. *AKR1C3* together with *LDLR* were also involved in “Retinoid metabolism and transport” and “Ovarian steroidogenesis”.

“Keap1-Nrf2 pathway” was enriched by heme oxygenase 1 (*HMOX1*) and glutamate-cysteine ligase regulatory subunit (*GCLM*) genes; *HMOX1* also contributed together with *TXNRD1* to “Oxidative stress” pathway enrichment and dominated as the only gene in the “Heme catabolic” pathway. *HMOX1* only contributed to the “Validated transcriptional targets of AP1 family members Fra1 and Fra2”.

Urokinase-type plasminogen activator (*PLAU*) and serpin peptidase inhibitor, clade B (ovalbumin), member 2 (*SERPINB2*) were genes contributing to over-representation of multiple pathways (“Dissolution of Fibrin Clot”, “Fibrinolysis Pathway”, “Plasminogen activating cascade”, “Blood clotting cascade”, “Blood coagulation” and together with *IL24* in “Senescence and autophagy”). *PLAU* itself further contributed to numerous pathways in cooperation with a variety of other genes such as *LDLR* and *BIK*, BCL2-interacting killer (“DNA damage response (only ATM dependent)”), *CTGF* (“amb2 Integrin signaling”), E2F transcription factor 2 (*E2F2*), fibroblast growth factor receptor 3 (*FGFR3*) and *HMOX1* (“MicroRNAs in cancer”).

*FGFR3* was the only gene contributing to “t(4;14) translocations of FGFR3” and together with *E2F2* was involved in “Bladder cancer”. Heat shock 27 kDa protein 1 (*HSPB1*) and stratifin (*SFN*) were involved in “p38 signaling mediated by MAPKAP kinases” pathway enrichment.

Different responses were observed after 24 h incubation: fewer genes were shared by all treatments and generally only several genes contributed to the deregulation of specific pathways (Table 3). Among top ranked pathways belonged ”Benzo[a]pyrene metabolism” with the contribution of *AKR1C2* and *AKR1C4*. These two genes also played a role in over-representation of other pathways such as ”Synthesis of bile acids and bile salts via 27-hydroxycholesterol”, ”Synthesis of bile acids and bile salts via 24 hydroxycholesterol”, ”Synthesis of bile acids and bile salts via 7alpha-hydroxycholesterol”, ”Synthesis of bile acids and bile salts”, ”Bile acid and bile salt metabolism”, ”Steroid hormone biosynthesis”, ”Metabolism of xenobiotics by cytochrome P450” and together with *TXNRD1* and 3-hydroxy-3-methylglutaryl-CoA synthase 1 (soluble) (*HMGCS1*) to ”Metabolism of lipids and lipoproteins”. *TXNRD1* was also important for ”thioredoxin pathway” and together with further contribution of kynureninase (*KYNU*) and interleukin 1 beta (*IL1B*) for ”Selenium pathway”*. COL7A1* and *COL8A1* were genes involved in deregulation of ”Genes encoding collagen proteins”, ”Assembly of collagen fibrils and other multimeric structures”, ”Collagen biosynthesis and modifying enzymes”, ”Collagen formation”. ”Protein digestion and absorption” was modulated due to the *COL7A1* and solute carrier family 3 (amino acid transporter heavy chain), member 2 (*SLC3A2*) action and ”ketone bodies metabolic” due to *HMGCS1*.

##### Analysis of Variance

One-way ANOVA was employed to discover differently modulated gene expression levels across all groups of treatment (NEXBTL100, B0, B30 and B100). After 4 h exposure, a significant alteration in expression levels of 99 genes was found. The vast majority of detected genes was distinctively modulated in response to NEXBTL100 compared to other treatments (Figure S1A). The functional annotation of the NEXBTL100-specific gene set revealed genes associated with the regulation of cell cycle. Genes involved in cell cycle (*MCM8*, *RBL1*, *RPS27*, *CDC25C*, *ENSA*, *ZWINT*) exhibited elevated expression levels, while other regulators were rather suppressed (*RAE1*, *CDKN2A*, *CCNB2*). The NEXBTL100 gene set further indicated the enhanced folding of actin by CCT/TriC (*CCT6A*, *TCP1*), purine metabolism (*ADSS*, *GART*) and repressed expression levels of genes participated in oxidative stress (*HMOX1*, *TXNRD1*, *GPX1*) or respiratory electron transport and mitochondrial function (*CYC1*, *UQCRFS1*, *NDUFV2*, *NDUFS3*).

ANOVA also revealed significant differences in expression levels of 152 genes across all groups of treatment upon 24 h incubation. We identified a distinctive pattern of modulated genes involved in the regulation of cell cycle (*KIF20A*, *AURKA*, *ACTR1A*, *UBA52*, *LMNA*, *CENPA*, *PSMC1*, *GINS2* or *MCM4*). Expression levels of these genes were almost exclusively elevated by NEXBTL100, while the lowest levels were detected upon B0 and B30 treatment. Expression levels of *RANBP1* and *TPX2*, genes involved in Ran-mediated regulation of mitotic spindle assembly were lowest upon B0 treatment compared to B30 displaying the highest potency to induce these genes (Figure S1B).

#### 2.4.3. Quantitative RT-PCR Validation of Selected Genes

**Figure 6:**
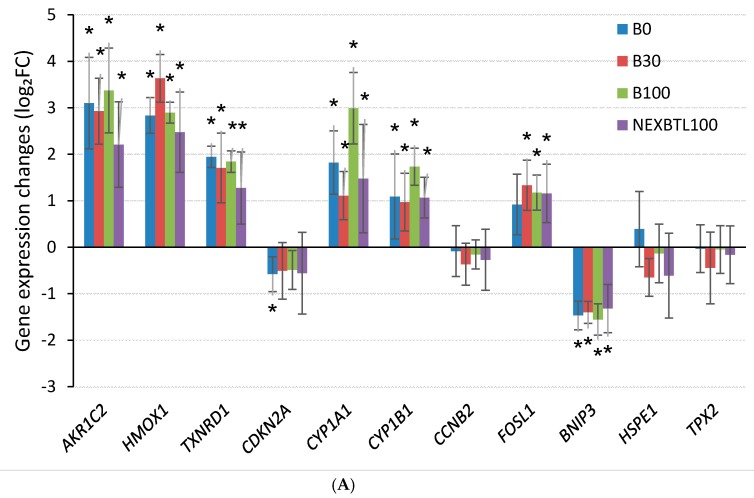

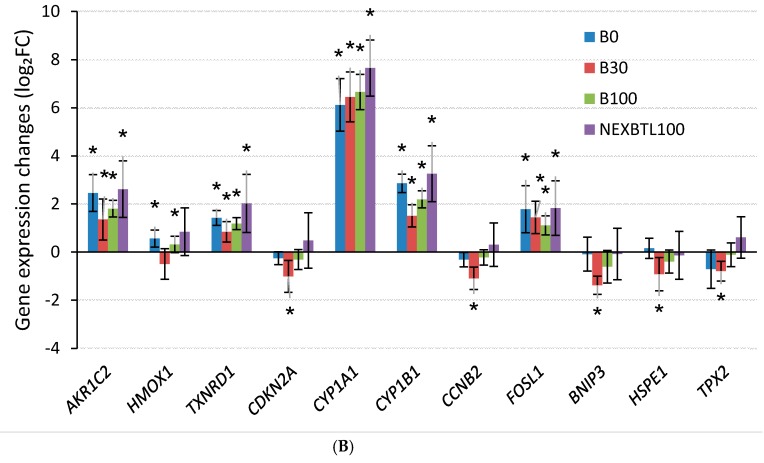
Quantitative RT-PCR verification of gene expression data obtained by microarray analysis. Eleven significantly deregulated genes were selected across all results and their gene expression levels were determined using qRT-PCR. Gene expression changes (log_2_ FC) were obtained by normalization to control samples (2^−ΔΔ*C*t^ method). Expression changes of selected genes upon: (**A**) 4 h exposure; and (**B**) 24 h exposure. * Indicates a statistically significant difference (*p*-value < 0.05).

For verification of gene expression changes in microarray analysis, qRT-PCR of eleven selected genes was performed (Figure 6). We focused on genes involved in processes and pathways related to the metabolism of xenobiotics (*AKR1C2*, *CYP1A1* and *CYP1B1*), activation of antioxidant defense (*HMOX1* and *TXNRD1*), AP-1 transcription, induction of plasminogen activator urokinase and modulation of cell adhesion (*FOSL1*), modulation of cell cycle (*CCNB2*), apoptosis (*CDKN2A* and *BNIP3*), regulation of spindle function (*TPX2*), endoplasmic reticulum stress and unfolded protein response (*HSPE1*).

## 3. Discussion

In the present study, we aimed to reveal a molecular signature of the toxicity induced by different organic compound mixtures extracted from diesel exhaust particles produced by diesel/renewable fuels in human lung BEAS-2B cells. A comparative analysis of gene expression changes was used to characterize the common mode of action underlying DEP extract exposure as well as specific effects of individual DEP extract treatments. 

Recent studies on the toxicity of different diesel and biodiesel exhaust particles suggest inconsistent data due to different running conditions of engines and experimental approaches [23,24]. Here, we used a standardized procedure providing the same conditions for particle collection, extraction and cell treatment thus enabling the accurate comparison of DEP extracts on gene expression changes. Although in vitro exposure to whole particles is more relevant in terms of simulation of real world conditions, many difficulties arising from intact particle collection, the characterization of particles and their behavior in culture medium may consequently complicate the interpretation of results. It has been documented that organic compounds bound to DEP are responsible to a large extent for its genotoxicity and other effects [12,25]. To the best of our knowledge, a study investigating effects of organic extracts from various fuels engine emissions on global gene expression changes in a model cell line system has not been published yet, so comparable data are not available. However, several authors reported the results of genotoxicity/mutagenicity of similar organic extracts. Bulky DNA adduct formation in a cellular calf thymus DNA system was consistently increased after treatment of the samples with extracts containing higher levels of PAHs [12,25]. In another study that investigated mutagenic properties of organic extracts of diesel and biodiesel fuels, higher PAHs content was associated with increased mutagenicity and bactericidal effects in *Salmonella typhimurium* TA98 and YG1041 strains [19]. These reports are in line with our observation that application of NEXBTL100 extract that contains lower levels of PAHs results in weaker biological response. This suggests that not only genotoxicity/mutagenicity, but also global gene expression changes are strongly affected by the presence of PAHs in the samples.

### 3.1. Common Cellular Response—4 h Cell Exposure

Despite the variability in chemical composition of individual DEP extracts, we revealed numerous genes and pathways altered in the same manner. Commonly deregulated genes following 4 h exposure were mostly involved in oxidative stress response and consequent events, such as activation of Nrf-2 and AP-1 transcription, antioxidant defense and DNA damage response.

The most significantly deregulated genes were *AKR1C2* and *AKR1C3*. A wide substrate specificity of these enzymes determines their implication in the metabolism of various exogenous and endogenous compounds such as steroids, sugars, carbonyls and others. They have a dual role in toxicity: under the control of Nrf-2 transcription factor they exhibit a detoxifying function by conversion of toxic aldehydic products [26] or catalyze the NADPH-dependent reduction of the *o*-quinone products to catechols and thus exacerbating ROS formation [27]. Induction of *AKR1C1*, *1C2* and *1C3* was also observed in BEAS-2B cells exposed to urban particulate matter in the study of Longhin et al., 2016 [28]. 

Polycyclic aromatic hydrocarbons and other organic compounds are capable of producing a substantial amount of ROS, which consequently lead to stabilization and activation of transcription factor Nrf-2 and induction of antioxidants and detoxifying enzymes [27]. Nrf-2 participates in the regulation of oxidant-stimulated functions, such as autophagy, inflammasome assembly, ER stress/UPR, mitochondrial biogenesis or stem cell regulation as well as protects against toxicity and chronic diseases in normal cells or through pharmacological interventions [29].

In our study, we observed elevated expression levels of *HMOX1*, *GCLM* and *TXNRD1* suggesting anti-oxidant response against ROS production. Surprisingly, we were not able to detect an increase in ROS production by carboxy-H_2_DCFDA assay. The same effect was reported in the study of Li et al., 2002 [30] where DEP extract treatment failed to induce DCF fluorescence in BEAS-2B, while the same treatment and detection method was effective in different cells. However, the authors confirmed the pro-oxidative potential by using a different method reflecting mostly production of superoxide radical. They also demonstrated a decrease of the GSH/GSSG ratio in BEAS-2B cells but not in THP-1 macrophages, as well as increased expression of *HMOX1*, pro-inflammatory cytokine *IL-8*, activation of JNK and decreased cell viability. In contrast, we observed no effect on reduced glutathione levels, suggesting effective replenishment of depleted GSH stores possibly due to the enhanced expression of *GLCM*. Moreover, other recent studies have demonstrated the pro-oxidative and pro-inflammatory effect in BEAS-2B and other cell lines upon exposure to DEP chemicals [31]. Importantly, the authors underscore the close relation of increasing oxidative potential of DEP extracts and the higher content of PAHs.

An excessive amount of ROS can further activate intracellular signaling cascades, including the mitogen-activated protein kinase. We detected significant upregulation of *HSPB1*, a protein chaperon involved in stress resistance and regulation of apoptosis. *HSPB1* maintains glutathione in its reduced form and decreases the amount of reactive oxygen species (ROS) produced in cells exposed to oxidative stress or tumor necrosis factor TNFα [32]. This could further support the hypothesis of a vigorous antioxidant response, which effectively neutralizes ROS and restores GSH levels.

ROS generation by DEP extracts arising from enzymatic metabolism of organic compounds as well as formation of reactive intermediates possibly cause DNA damage with consequent cell cycle arrest, senescence and cell death. Our data strongly suggest activation of p53 signaling due to the modulation of genes involved in “DNA damage response (ATM induced) pathway” (*BIK*, *LDLR*, *PLAU*). We also observed the induction of *SFN*, a direct p53 effector, suggesting cell cycle arrest in response to DNA double strand breaks. Suppression of *CCNB2* by all extract treatments, as confirmed by qRT-PCR, may indicate inhibition of cell cycle. It has been shown that the activity of *CCNB2*, a key factor essential for transition from G2 to mitosis, is under the control of p53 which represses transcription of *CCNB2* and causes arrest in G2 phase upon DNA damage [33]. Elevated expression of *GLIPR1*, another p53 target, could further contribute to increased ROS production, promote cell cycle arrest and apoptosis, as observed in prostatic cancer cells [34]. On the other hand, pro-apoptotic *BNIP3* was significantly downregulated by all treatments. This suppression of *BNIP3* might be mediated by p53 acting in favor of the protection against hypoxia-induced cell death as documented in Feng et al. [35]. Suppression of *CDKN2A*, a stabilizer of p53 protein, *BIK* and *BNIP3* may suggest the suppression of p53 activity and anti-apoptotic response of the cells. *FOSL1* induction might be related to AP-1 transcription, induction of plasminogen activator urokinase (*PLAU*) and modulation of cell adhesion [36]. Other genes contributing to DNA damage response indicated cellular senescence and/or autophagy (*PLAU*, *SERPINB2*).

The role of plasminogen activator (*PLAU*) and its inhibitor (*SERPINB2*) in senescence is not fully understood; however, the study of West et al. [37], described alterations in plasminogen activator activity during replicative senescence leading to the disruption of extracellular matrix maintenance with possible deleterious consequences on tissue homeostasis. According to our results, in the study of Longhin et al. [28], the authors also found a strong induction of *SERPINB2* and *PLAU* in BEAS-2B cells exposed to whole airborne particles. Exposure to air pollution may cause acute exacerbation of idiopathic pulmonary fibrosis [38]. Interestingly, enhanced expression of *PLAU* and *SERPINB2* and simultaneous suppression of *CTGF*, a growth factor promoting the fibrosis, observed in our study, may suggest the anti-fibrotic response. Plasminogen activator and plasminogen activator-inhibitor are components of the plasminogen activation system, which has been implicated in fibrosis reduction [39]. Similarly, a defensive role of Nrf2 target, *TXNRD1*, against fibrosis has also been described [40]. Moreover, activation of peroxisome proliferator-activated receptor α (PPARα) participating in the processes of both physiological and toxicological response to various endogenous or exogenous substances may also protect against lung fibrosis [41]. The multiple regulatory role of PPARα in various processes related to oxidative stress, lipid metabolism and inflammation has been documented [42]. 

### 3.2. Differential Response Detected by Analysis of Variance—4 h Cell Exposure

A vast majority of genes detected by ANOVA among all treatments were distinctively modulated in response to NEXBTL100 treatment and exhibited more than 1.5-fold change in expression levels compared to a group median involving other gene sets (B0, B30 and B100). Most of the NEXBTL100-specific genes were involved in DNA replication, cyclin B2 related events and entry into mitosis. Elevated expressions of *MCM8*, *RBL1*, *RPS27*, *CDC25C*, *ENSA* and *ZWINT* may suggest deregulation towards enhanced proliferation compared to other DEP extract treatment. Accordingly, repression of *RAE1*, a mitotic checkpoint regulator, as well as *CDKN2A*, a tumor suppressor that inhibits G1/S transition and establishes cell cycle arrest, could further support the hypothesis of enhanced proliferative potential of NEXBTL100 extract.

Analysis of top-ranked over-represented pathways specific for each DEP extract treatment showed that “Keap-Nrf2 pathway” and “Glutathione biosynthesis pathway” were the most significantly affected by NEXBTL100 4 h exposure. However, NEXBTL100 induced the lowest levels of *HMOX1*, *TXNRD1* and *GPX1* suggesting modest anti-oxidative response, possibly caused by the lowest production of ROS. It should be stressed that changes were not significant compared to unexposed controls. However, subtle changes in gene expression levels detected by ANOVA may also contribute to distinctive gene expression patterns of individual DEP extracts treatments. These results indicate that NEXBTL100 induced the weakest oxidative stress response and DNA damage and exhibited elevated expression of genes possibly contributing to increased proliferation compared to other treatments.

On the other hand, the list of the most over-represented pathways modulated by B0, B30 and B100 was similar to each other and involved “Benzo[a]pyrene metabolism” and similar pathways (with *AKR1C2* and *CYP1B1* being the most contributing deregulated genes) and “Plasminogen activating cascade” (with a significant contribution of *SERPINB2*, *PLAU*, *PLAT*). Additionally, B100 extract, which contained the highest concentrations of carcinogenic PAHs, also affected most significantly the “Cell cycle”, “p38 signaling” and “Senescence and autophagy” pathways (data not presented here).

### 3.3. Common Cellular Response—24 h Cell Exposure

The major toxic response following 24 h exposure to all DEP extracts was the metabolic activation of PAHs. The key enzymes contributing to modulation of “Benzo[a]pyrene metabolism” and “Metabolism of xenobiotics by cytochrome P450” as well as numerous other pathways were *AKR1C2* and *AKR1C4*. AKRs participate in *o*-quinone pathway by conversion of PAH-diols into redox active PAH *o*-quinones, but also facilitate the redox cycling of the PAH *o*-quinones to catechols. Catechols are able to conjugate with a wide range of conjugating enzymes. Conjugation terminates the redox cycling, eliminates formation of electrophilic products and prevents formation of covalent adducts [43]. We also confirmed significantly elevated expression of other important PAH-metabolic enzymes *CYP1A1* and *CYP1B1* by qRT-PCR, although microarray data did not indicate a significant increase, possibly due to the high variability among replicates. CYP enzymes are involved in the formation of *trans*-dihydrodiols, the first step of PAH activation and also in the consequent event when dihydrodiols are converted into diol-epoxides, which can covalently bind to DNA and form persistent DNA adducts. The competing role of CYP and AKR enzymes in the metabolic activation of PAH-diols has been observed in human bronchoalveolar cell extracts [44].

The induction of CYP enzymes is dependent on the activation of AhR. Our recent data of AhR-mediated activity performed in human AZ-AhR cells (Stable HepG2 Luciferase Reporter Cell Line) by CALUX assay, suggest similar TEQ values for B0, B30 and B100 extracts and lower TEQ value for NEXBTL100 extract (see Figure S2), which accordingly reflected a lower PAH content. The similar trend could be also expected for BEAS-2B cell line. Our previous findings indicate that concentration of PAHs in a mixture of organic compounds extracted from reference urban dust particulate matter is higher [45] than in similar organic extracts from standard reference diesel exhaust particle material [25]. Therefore, also AhR-mediated activity of DEP extracts was lower. These results are in line with our present findings where only partial induction of CYP1A1 and CYP1B1 mRNA confirmed the low activation of the AhR-dependent gene expression. Interestingly, a recent study of Palkova et al., 2015 [25] analyzing the toxicity of organic fraction extracted from reference material of diesel exhaust particles (SRM 1650b) consistently evidenced its high AhR-inducing activity and potency to induce metabolic enzymes, trigger DNA damage response and disrupt cell cycle progression and proliferation in rat lung epithelial cells RLE-6TN and rat liver epithelial cells WB-F344 in the concentration range of 100–1000 and 50–1000 µg/mL, respectively, suggesting a higher potency to metabolize PAHs compared to other cell lines.

Interestingly, a higher metabolic rate of organic compounds probably caused the interference with WST-1 assay and gave false positive results of “enhanced” proliferation upon low dose exposures to DEP extracts. Since WST-1 assay is based on conversion of tetrazolium dye by mitochondrial dehydrogenases, the results obtained by WST-1 assay more likely imply enhanced metabolic activity of cells due to the effect of organic compounds and not increased number of cells. 

### 3.4. Differential Response Detected by Analysis of Variance—24 h Cell Exposure

Similar to 4 h incubation, ANOVA also revealed distinct gene expression patterns following 24 h incubation across all treatments. Functional annotation of genes identified by ANOVA revealed that the most distinctive signature was induced by NEXBTL100 extract treatment. Specifically, we found a marked difference in expression levels of genes associated with regulation of chromosome segregation and cytokinesis. Among them, *AURKA* is critical for the proper formation of mitotic spindle [46], while *CENPA* controls kinetochore assembly and chromosome segregation [47] and *KIF20A*, a mitotic kinesin, is required for chromosome passenger complex (CPC)-mediated cytokinesis [48]. It has been demonstrated that p53 acts as a negative regulator of *AURKA* activity and reduces its expression level after DNA damage [49]. Deletion of *AURKA* may consequently cause cell cycle arrest. *AURKA* requires a number of co-factors for its activation such as microtubule associated protein *TPX2* and GTPase Ran. Ran releases *TPX2* to bind and activate *AURKA* by changing its conformation, stimulating its autophosphorylation and targeting it to spindle microtubules at the pole [50]. Importantly, overexpression of *AURKA* and *TPX2* has been linked to tumor development at different levels [51]. We confirmed the elevated expression of *TPX2* upon NEXBTL100 extract treatment by RT-qPCR, while other treatments rather slightly suppressed gene expression level. *TPX2* is an essential regulator of spindle function and may indicate the possible negative effect of DEP extracts (excepting NEXBTL100) on mitosis and generally on cell cycle progression [52].

### 3.5. Study Limitations

Although our study represents a comprehensive analysis of toxic effects of organic extracts from various fuels engine emissions, there are several limitations that should be acknowledged.

The first limitation is related to the nature of the samples used for in vitro tests. The results reported here are based on exposing the cells to the extracts from comparable masses of particles. The results therefore represent the “quality” of the particles, expressed as some metric of effect per mg of particulate matter, a metric suitable for attempting to comprehend the mechanisms of the effects of particles on human health. For realistic evaluation of fuels, however, the effects of fuels should be compared based on distance driven, amount of useful work performed by the engine, fuel consumed, or similar metric. Since the total mass of particles emitted varied among the fuels (see Figure 1B), and the effects are believed to be nonlinear, the results do not necessarily represent the realistic effects of a fuel substitution on human health. In addition, due to the anticipated nonlinearity of effects with respect to dose, expressing the effects per kg of fuel would be of limited use. The real exposure is not to raw, undiluted exhaust, and the effective dilution ratio (reciprocal of the fraction of raw exhaust in inhaled air) considerably varies with conditions (mainly proximity of vehicles to population and atmospheric conditions). Evaluation of “realistic” fuel effects would therefore necessitate an arbitrary and difficult to justify assumption of a certain dilution ratio.

Another limitation is the fact, that we used organic extracts rather than exhaust particles to investigate biological effects of various fuel emissions. Although particles mediate some unique effects, particularly oxidative stress induction that cannot be directly mimicked by extracts, organic fraction contains PAHs, i.e., compounds with highest genotoxic activity and most immediate impact on human health. It should be also noted that the use of organic extracts increase the bioavailability of PAHs compared to whole particles experiments therefore the results may overestimate the effects of DEP-associated PAHs. Oxidative stress induction measured in our study yielded mostly negative results, although this may be caused by technical limitations of the test methods. 

Finally, although gene expression profiling is a robust tool that covers simultaneously whole-genome gene expression changes, it still measures a single endpoint (mRNA levels). To get more insight into the mechanism of action of tested toxicant(s), gene expression profiling experiments should be integrated into larger studies examining multiple end-points at the molecular, cellular, tissue, and physiological levels in the context of the whole organism. Therefore, our study does not aim to reveal the precise mechanism of action (e.g., cell cycle regulation or other fundamental cellular processes) but rather provides new testable hypothesis that could be subsequently confirmed by further experiments.

## 4. Materials and Methods

### 4.1. Chemicals and Biochemicals

Bronchial Epithelial Basal Medium and BEGM™ BulleKit™ were purchased from Lonza (Basel, Switzerland); Human bronchial epithelial cells BEAS-2B from ATCC (Manassas, VA, USA); *tert*-butyl hydroperoxide, Glutathione Fluoriometric Assay Kit, NaHCO_3_, HEPES, and non-essential amino acids were obtained from Sigma-Aldrich (St. Louis, MO, USA); WST-1 Proliferation Assay and High Fidelity cDNA synthesis Kit from Roche (Mannheim, Germany); 5-(and-6)-carboxy-2′,7′-dichlorodihydrofluorescein diacetate and Hank’s Balanced Salt Solution from Thermo Fisher Scientific (Waltham, MA, USA); NucleoSpin RNA II from Macherey-Nagel (Düren, Germany); Illumina Human-HT12 v4 Expression BeadChips were from Illumina (San Diego, CA, USA); Illumina TotalPrep RNA Amplification Kit from Ambion (Austin, TX, USA); RT-qPCR master mix, PerfectProbe assays and geNorm Reference Gene Selection Kit from Primerdesign (Southampton, UK); Dulbecco’s Modified Eagle’s Medium and Gentamicin Sulfate from Gibco (Paisley, UK) and Luciferase Assay kit from BioThema (Handen, Sweden).

### 4.2. Test Vehicle and Exhaust Particles Collection

Engine tests and particle collection were performed on a transient engine dynamometer test stand at the laboratories of the Czech Technical University in Prague in VTP Roztoky. An Iveco Tector 5.9 L, 176 kW engine commonly used in the EU (and similar in design to Cummins ISB engine used widely in the U.S.) was utilized as a representative of a common diesel engine of a small truck. The engine was operated without after treatment to represent the type of engines responsible for the majority of particulate matter in the air. Exhaust gases were transferred into a full-flow dilution tunnel where the gases were mixed with fresh air filtered through active carbon and high-efficiency particulate air (HEPA) filters. A constant volume sampler system kept the flow rate through the dilution tunnel to 50 m^3^/min. DEP samples were taken from the dilution tunnel by two high volume Ecotech 3000 samplers with installed impactor (cut off particle diameter 2.5 µm). The sample flow rate was set to 67.8 m^3^/h. The samplers were normally used for atmospheric sampling and were modified for use with diluted exhaust and subsequently augmented with an auxiliary three-stage blower to increase the filter capacity. Borosilicate filters (8” × 10”, Emfab, TX40HI20-WW, Pall, Port Washington, NY, USA) were used to collect hundreds of milligrams of DEP for the extraction procedure and toxicity testing. The World Harmonized Transient Cycle (WHTC) used for type-approval of heavy-duty engines was chosen as a test cycle. This cycle was run once with a cold start and nine times with a hot start (it should be noted the engine was not fully warmed up after one WHTC). Four different fuels were used: pure diesel (B0, Cepro, a.s., Prague, Czech Republic), neat fuel-grade biodiesel (B100, methylesters of rapeseed oil, Cepro, a.s., Prague, Czech Republic), a blend of 70% B0 and 30% B100 (*v*/*v*) mixed from B0 and B100, and pure hydrotreated vegetable oils and waste animal fats (NEXBTL100, Neste Oil, Espoo, Finland). Before sampling, the engine ran 3 WHTC in order to adapt to a new fuel. The engine was then left overnight at 23 °C as a preconditioning for the cold start WHTC to prepare it for the sampling with a cold start the next day. DEPs collected within 10 WHTCs for every fuel was used for chemical and toxicological analysis.

### 4.3. Particle Characterization and Chemical Analysis

Particle size distributions were measured using a fast mobility particle sizer (EEPS, Engine Exhaust Particle Sizer, model 3090, TSI, St. Paul, MN, USA), coupled to a rotating disc microdiluter (model MD-11, Matter Engineering Inc., Wohlen, Switzerland) set to a dilution ratio of 150:1, with the dilution head heated to 150 °C. Fuel consumption was recorded using an AVL 735S Fuel Mass Flow Meter (AVL, Graz, Austria).

Organic compounds from diesel exhaust particles produced by different fuels (B0, B30, B100 and NEXBTL100 were extracted with 70 mL of dichloromethane in an automated extraction apparatus (Behr EF, BEHR, Stuttgart, Germany) for 4 h (3 h in boiled solvent and 1 h in condensed solvent). Requested aliquots of the extract were re-dissolved in required volume of acetonitrile for HPLC/DAD and LC/MS-MS; and in dimethylsulfoxide (DMSO) for biological in vitro assays. Identification of compounds was performed by comparison of their retention times with authentic standards. The method of external standardization was used for quantification of contaminants. The accuracy and precision of the analytical methods was determined by analyzing of the standard reference material SRM 1650b (Diesel Particulate Matter, NIST, Gaithersburg, MD, USA). Further details on chemical analysis are described elsewhere [45].

### 4.4. Cell Cultures and Exposure Conditions

Human bronchial epithelial cells BEAS-2B were cultured in Bronchial Epithelial Basal Medium (BEBM) supplemented with the standardized set of growth factors provided by the manufacturer (BEGM™ BulleKit™). All cultivation flasks and plates were coated with BEBM containing fibronectin, collagen and bovine serum albumin. All tested DEP extracts were diluted in complete BEBM and cells were incubated in triplicates for 4 h or 24 h, respectively. Control cells were incubated with DMSO extracts from blank filters. Cell cultures and cell-based assays were maintained in a humidified atmosphere with 5% CO_2_ at 37 °C.

### 4.5. Cytotoxicity

To assess the viability of cells, the WST-1 Proliferation Assay was performed 24 h after exposure to DEP extracts following the manufacturer’s protocol. BEAS-2B were cultivated in 96-well plates 24 h before treatment in a density of 7500 cells per well. For dose–response curve and threshold cytotoxicity assessment, cells were incubated in 18 concentrations (22–1000 μg/mL) of each DEP extract obtained by serial dilution in BEGM. Positive controls were incubated with 0.1% Triton X-100 and negative controls with complete medium.

### 4.6. Cell Culture Conditions, RNA Isolation and Quality Control

For gene expression profiling, cells suspended in complete BEBM were seeded in pre-coated Petri dishes (surface area 22.1 cm^2^). Cell seeding density was 27,000 and 18,000 cells/cm^2^ for 4 and 24 h exposure period, respectively. After 44 hours, media was removed, cells were washed with PBS and 3 mL of fresh dilutions of DEP extracts in complete BEBM (50 μg/mL) and controls (complete BEBM and 0.1% DMSO) were added to the cells. Triplicates were prepared for each DEP extract and control. Cells were incubated in humidified atmosphere with 5% CO_2_ at 37 °C for 4 and 24 h.

Total RNA from lysed BEAS-2B cells was obtained using NucleoSpin RNA II according to the manufacturer’s instructions. RNA concentration was quantified with a Nanodrop ND-1000 Spectrophotometer (Thermo Fisher Scientific, Waltham, MA, USA). The integrity of RNA was assessed using an Agilent 2100 Bioanalyzer (Agilent Technologies Inc., Santa Clara, CA, USA). All samples had an RNA Integrity Number (RIN) greater than 9. Isolated RNA was stored at −80 °C until processing.

### 4.7. Microarray Analysis

Illumina Human-HT12 v4 Expression BeadChips were used to generate gene expression profiles. Biotinylated complementary RNA (cRNA) was prepared from 200 ng of total RNA using the Illumina TotalPrep RNA Amplification Kit (Thermo Fisher Scientific, Waltham, MA, USA). Next, 1000 ng of biotinylated cRNA targets was hybridized to the beadchips. The steps of hybridization and the subsequent washing, staining and drying of the beadchips were performed according to standard instructions from Illumina. The hybridized beadchips were then scanned on the Illumina iScan and bead level data were summarized by Illumina GenomeStudio Software v2011.1 (Illumina Inc., San Diego, CA, USA).

### 4.8. Real-Time Quantitative PCR (RT-qPCR) Verification

One microgram of RNA from each sample was used for complementary DNA (cDNA) synthesis using the Transcriptor High Fidelity cDNA synthesis Kit (Roche, Basel, Switzerland). cDNA synthesis was performed twice for each sample to obtain a technical replicate. The original protocol was modified by using 2.5 μM oligo(dT) and 10 μM random hexamers for priming in a 20 μL reaction volume. cDNA synthesis was run according to the following conditions: 30 min at 55 °C and 5 min at 85 °C. Quantitative PCR measurements were performed using the 7900HT Fast Real-Time PCR System (Applied Biosystems, Carlsbad, CA, USA). Each RT-qPCR reaction was carried out in a final volume of 14 μL containing 3.5 μL of diluted cDNA, 2.8 μL of water and 7 μL of master mix. To determine the level of each target gene, 0.7 μL of a specifically designed assay (Custom designed real-time PCR assay with Double-Dye probe, Primerdesign, Eastleigh, UK) was added to the reaction mixture. Cycling conditions were: 2 min at 95 °C followed by 40 cycles of amplification (10 s at 95 °C and 60 s at 60 °C). The baseline and threshold values of RT-qPCR experiments raw data were assessed with SDS Relative Quantification Software version 2.3 (Applied Biosystems, Waltham, MA, USA) to determine Ct values. Expression levels of target genes were normalized to the reference genes (*B2M* and *ACTB*). Reference genes were selected according to the stability of gene expression during experimental conditions using the geNorm Reference Gene Selection Kit. Relative changes in normalized gene levels were calculated using the 2^−ΔΔ*C*t^ method [53]. The sequences of primers used in RT-qPCR are shown in Table S2.

### 4.9. Quantification of Intracellular ROS

The intracellular ROS levels upon DEP extracts treatments were detected by using 5-(and-6)-carboxy-2’,7’-dichlorodihydrofluorescein diacetate (carboxy-H_2_DCFDA). Cells were plated into black 96-well plates one day before treatment in the density of 30,000 cells per well and each measurement was performed using a cell triplicate. Before treatments, cells were loaded with Hank’s Balanced Salt Solution containing 40 μM carboxy-H_2_DCFDA for 30 min. After dye loading, cells were washed two times with BEGM and incubated with 50 μg/mL of each DEP extract or 250 μM of *tert*-butyl hydroperoxide as a positive control for 4 h. Fluorescence was measured with a SpectraMax M5 plate reader (Molecular Devices, Sunnyvale, CA, USA) with an excitation and emission wavelength of 494 nm and 525 nm, respectively.

### 4.10. Quantification of Glutathione Levels

The total amount of reduced glutathione (GSH) in the cell cultures exposed to 50 μg/mL of DEP extracts for 4 h was quantified with a Glutathione Fluorimetric Assay Kit according to the manufacturer’s recommendations for a 96-well plate format. Absorbance was measured by a SpectraMax M5 plate reader at 494 nm. Concentrations of reduced GSH were calculated using a standard calibration curve; positive controls were treated with staurosporine (1 μg/mL). Each measurement was performed using a cell triplicate.

### 4.11. Statistical Analysis

Gene expression levels were compared with control BEAS-2B cell cultures treated with extract from blank filter only. Bead summary data were imported into R statistical environment (https://www.r-project.org/) and normalized using the quantile method in the Lumi package [54]. Only probes with a detection *p*-value < 0.01 in more than 50% of arrays were included for further analyses. Differential gene expression was analyzed in the Limma package using the moderated *t*-statistic. A linear model was fitted for each gene given a series of arrays using lmFit function [55]. A multiple testing correction was performed using the Benjamini and Hochberg method. To analyze lists of significantly deregulated genes after DEP extract treatments (cut-off *p*-value < 0.05, fold change > 1.5, or < 0.6), a ToppFun tool was used [56]. Functional analysis identified numerous over-represented terms in several categories; pathways as a functional category were considered for the analysis.

The detection of differential gene expression was performed using the parametric one-way ANOVA test. We employed the R Stats package [57] implementation using the design described in [58]. The *p*-values were adjusted for multiple comparisons using the BH method [59]. The Tukey’s “Honest Significant Difference” method [60] served as a “post-hoc” test. The data of ROS generation, reduced glutathione levels and expression of selected genes verified by RT-qPCR were expressed as mean values ± S.D. To detect significant changes between means of treated groups and the control group, the two-tail Student’s *t*-tests were used.

## 5. Conclusions

In conclusion, equal concentrations of organic extracts of diesel and biodiesel exhaust particles induced similar molecular response in BEAS-2B cells, although specific gene expression patterns were also observed. Following 4 h exposure, we found altered processes related to oxidative stress response (mostly antioxidant defense activation only), suppression of pro-apoptotic stimuli, regulation of cell cycle and plasminogen activating cascade. NEXBTL100 extract distinctively exhibited a modest antioxidant response, suggesting weaker stress response probably due to the less harmful nature of the organic compounds compared to other extracts. The key common events after 24 h incubation were metabolism of lipids and xenobiotics, including PAHs, and oxidative stress-induced pathways; however, no increase in ROS production was detected. Importantly, no pro-inflammatory genes (with the exception of *IL24*) and pathways were affected, cancer-related pathways were modulated minimally and only a low AhR-mediated activity was found at sub-cytotoxic concentration of DEP extracts. Biological effects of induction of plasminogen activating cascade (*SERPINB2*, *PLAU* and other genes) in human bronchial cells should be further studied.

This study used the toxicogenomic approach to identify biological processes and pathways affected by organic components of diesel exhaust particles produced by four commonly used fuels. Although NEXBTL100 extract exhibited the weakest toxic response, our findings indicate subtle differences in overall toxic effects induced by different diesel or alternative fuels’ DEPs, or their blends when the equal amount of particles and qualitative composition of organic compound mixtures was considered. Other factors, such as fuel consumption, total amount of produced particles or particle size distribution may influence substantially the resulting toxic effects.

## Figures and Tables

**Table 1:** Results of chemical analysis.

**PAHs**				
**Compound (ng/mg DEP)**	**B0**	**B30**	**B100**	**NEXBTL100**
Fluoranthene	41.9	110	117	39.4
Pyrene	67.7	152	144	43.1
Benz[a]anthracene *	6.6	9.8	26.1	5.0
Chrysene *	5.1	6.0	12.5	1.0
Benzo[b]fluoranthene *	1.2	1.4	7.0	0.3
Benzo[k]fluoranthene *	n.d.	n.d.	2.6	n.d.
Benzo[a]pyrene *	0.6	0.9	1.7	1.2
Benzo[g,h,i]perylene	2.2	2.8	3.8	2.0
Dibenz[a,h]anthracene *	n.d.	n.d.	n.d.	n.d.
Indeno[1,2,3-cd]pyrene *	0.7	0.8	1.8	n.d.
4H-Cyclopenta[d,e,f]phenathrene	2.8	3.8	4.3	9.4
Benzo[c]phenanthrene	1.9	3.8	4.0	1.3
Benzo[j]fluoranthene	2.4	2.8	6.4	2.0
Benzo[e]pyrene	0.7	0.6	3.6	0.3
Triphenylene	9.0	3.6	4.0	1.1
Benzo[c]chrysene	n.d.	n.d.	0.2	n.d.
Coronene	0.8	0.7	0.7	0.6
**Methylated PAHs**				
**Compound (ng/mg DEP)**	**B0**	**B30**	**B100**	**NEXBTL100**
1-Methylpyrene	5.1	7.2	3.9	1.4
2-Methylpyrene	8.5	11.0	8.0	2.9
4-Methylpyrene	10.0	13.4	12	3.7
1-Methylchrysene	0.3	0.3	1.2	n.d.
7-Methylbenz[a]anthracene	0.3	n.d.	n.d.	0.2
**Oxygenated PAHs**				
**Compound (ng/mg DEP)**	**B0**	**B30**	**B100**	**NEXBTL100**
1,8-Naphthalic Anhydride	25.4	29.5	29.7	19.8
Phenanthrene-9,10-dione	1.3	0.8	0.8	0.5
9H-Fluoren-9-one	4.3	2.8	1.7	4.7
Anthrone	n.d.	n.d.	n.d.	n.d.
Anthracene-9,10-dione	2.7	4.5	4.0	2.6
7H-Benz[d,e]anthracene-7-one	1.4	1.5	2.4	0.28
9-Hydroxybenzo[a]pyrene	0.06	0.07	0.09	0.07
Benz[a]anthracene-7,12-dione	0.02	0.04	0.15	0.01
3-Hydroxybenzo[a]pyrene	0.05	0.05	0.10	0.02
**Nitrated PAHs**				
**Compound (pg/mg DEP)**	**B0**	**B30**	**B100**	**NEXBTL100**
1-Nitropyrene	314	539	1504	126
2-Nitropyrene	25.0	12.0	23.0	n.d.
4-Nitropyrene	18.0	16.0	44.0	10.0
3-Nitrofluoranthene	7.0	16.0	41.0	2.0
**Dinitrated PAHs**				
**Compound (pg/mg DEP)**	**B0**	**B30**	**B100**	**NEXBTL100**
1,3-Dinitropyrene	1.6	2.4	17.7	0.4
1,6-Dinitropyrene	1.2	5.6	65.0	n.d.
1,8-Dinitropyrene	0.9	4.6	48.0	n.d.

* Human carcinogens (IARC). PAHs, polycyclic aromatic hydrocarbons; DEP, diesel exhaust particles; n.d., not detectable.

**Table 2:** Top ranked over-represented pathways shared by all DEP extract treatments following 4 h incubation. Functional enrichment was performed using ToppFun tool integrating numerous annotation databases. Significant upregulation resp. downregulation of genes: ↑↓.

Name	Genes from Input
”Benzo[a]pyrene metabolism”, ”Synthesis of bile acids and bile salts via 27-hydroxycholesterol”, ”Synthesis of bile acids and bile salts via 24-hydroxycholesterol”, ”Synthesis of bile acids and bile salts via 7α-hydroxycholesterol”, ”Synthesis of bile acids and bile salts”, ”Bile acid and bile salt metabolism”	↑ *AKR1C3*, *AKR1C2*
”Metabolism of lipids and lipoproteins”	↑ *TXNRD1*, *GLIPR1*, *AKR1C3*, *AKR1C2*; ↓ *LDLR*, *CTGF*
”PPARA Activates Gene Expression”, ”Regulation of Lipid Metabolism by Peroxisome proliferator-activated receptor alpha (PPARalpha) “	↑ *TXNRD1*, *GLIPR1*; ↓ *CTGF*
”Retinoid metabolism and transport, Ovarian steroidogenesis”	↑ *AKR1C3*; ↓ *LDLR*
”Oxidative Stress”	↑ *TXNRD1*, *HMOX1*
”Keap1-Nrf2 Pathway”	↑ *HMOX1*, *GCLM*
”Validated transcriptional targets of AP1 family members Fra1 and Fra2”	↑ *PLAU*, *HMOX1*
”heme catabolic”	↑ *HMOX1*
”Dissolution of Fibrin Clot”, Fibrinolysis Pathway”, ”Plasminogen activating cascade”, ”Blood Clotting Cascade”, ”Blood coagulation”	↑ *PLAU*, *SERPINB2*
”Senescence and Autophagy”	↑ *IL24*, *PLAU*, *SERPINB2*
”amb2 Integrin signaling”	↑ *PLAU*; ↓ *CTGF*
”DNA damage response (only ATM dependent) “	↑ *PLAU*, *BIK*; ↓ *LDLR*
”intrinsic apoptotic”	↑ *BIK*; ↓ *BNIP3*
”t(4;14) translocations of FGFR3”	↑ *FGFR3*
“Bladder cancer”	↑ *FGFR3*; ↓ *E2F2*
”MicroRNAs in cancer”	↑ *PLAU*, *FGFR3*, *HMOX1*; ↓ *E2F2*
”p38 signaling mediated by MAPKAP kinases”	↑ *HSPB1*, *SFN*

**Table 3:** Top ranked over-represented pathways shared by all DEP extract treatments following 24 h incubation. Significant upregulation resp. downregulation of genes: ↑↓.

Name	Genes from Input
”Benzo[a]pyrene metabolism”, ”Synthesis of bile acids and bile salts via 27-hydroxycholesterol”, ”Synthesis of bile acids and bile salts via 24-hydroxycholesterol”, ”Synthesis of bile acids and bile salts via 7α-hydroxycholesterol”, ”Synthesis of bile acids and bile salts”, ”Bile acid and bile salt metabolism”, ”Steroid hormone biosynthesis”, ”Metabolism of xenobiotics by cytochrome P450”	↑ *AKR1C4*, *AKR1C2*
”Metabolism of lipids and lipoproteins”	↑ *TXNRD1*, *AKR1C4*, *AKR1C2*; ↓ *HMGCS1*
”ketone bodies metabolic”	↓ *HMGCS1*
”Selenium Pathway”	↑ *TXNRD1*, *IL1B*, *KYNU*
”thioredoxin pathway”	↑ *TXNRD1*
”Genes encoding collagen proteins”, ”Assembly of collagen fibrils and other multimeric structures”, ”Collagen biosynthesis and modifying enzymes”, ”Collagen formation”	↑ *COL7A1*; ↓ *COL8A1*
”Protein digestion and absorption”	↑ *COL7A1*, *SLC3A2*

## Supplementary Materials

Supplementary materials can be found at www.mdpi.com/1422-0067/17/11/1833/s1.

## References

[1] Aatola H., Larmi M., Sarjovaara T., Mikkonen S. (2009). Hydrotreated vegetable oil (HVO) as a renewable diesel fuel: Trade-off between nox, particulate emission, and fuel consumption of a heavy duty engine. SAE Int. J. Engines.

[2] Moser B.R. (2014). Impact of fatty ester composition on low temperature properties of biodiesel–petroleum diesel blends. Fuel.

[3] Kim D., Kim S., Oh S., No S.-Y. (2014). Engine performance and emission characteristics of hydrotreated vegetable oil in light duty diesel engines. Fuel.

[4] Omidvarborna H., Kumar A., Kim D.-S. (2016). Variation of diesel soot characteristics by different types and blends of biodiesel in a laboratory combustion chamber. Sci. Total Environ..

[5] Prokopowicz A., Zaciera M., Sobczak A., Bielaczyc P., Woodburn J. (2015). The effects of neat biodiesel and biodiesel and HVO blends in diesel fuel on exhaust emissions from a light duty vehicle with a diesel engine. Environ. Sci. Technol..

[6] Rakopoulos D.C., Rakopoulos C.D., Giakoumis E.G. (2015). Impact of properties of vegetable oil, bio-diesel, ethanol and n-butanol on the combustion and emissions of turbocharged hddi diesel engine operating under steady and transient conditions. Fuel.

[7] Singh D., Subramanian K.A., Singal S.K. (2015). Emissions and fuel consumption characteristics of a heavy duty diesel engine fueled with hydroprocessed renewable diesel and biodiesel. Appl. Energy.

[8] Woo C., Kook S., Hawkes E.R., Rogers P.L., Marquis C. (2016). Dependency of engine combustion on blending ratio variations of lipase-catalysed coconut oil biodiesel and petroleum diesel. Fuel.

[9] Xue J., Grift T.E., Hansen A.C. (2011). Effect of biodiesel on engine performances and emissions. Renew. Sustain. Energy Rev..

[10] Millo F., Debnath B.K., Vlachos T., Ciaravino C., Postrioti L., Buitoni G. (2015). Effects of different biofuels blends on performance and emissions of an automotive diesel engine. Fuel.

[11] Lapuerta M., Armas O., Rodríguez-Fernández J. (2008). Effect of biodiesel fuels on diesel engine emissions. Prog. Energy Combust. Sci..

[12] Vojtisek-Lom M., Pechout M., Dittrich L., Beranek V., Kotek M., Schwarz J., Vodicka P., Milcova A., Rossnerova A., Ambroz A. (2015). Polycyclic aromatic hydrocarbons (PAH) and their genotoxicity in exhaust emissions from a diesel engine during extended low-load operation on diesel and biodiesel fuels. Atmos. Environ..

[13] Tang S., LaDuke G., Chien W., Frank B.P. (2016). Impacts of biodiesel blends on pm2.5, particle number and size distribution, and elemental/organic carbon from nonroad diesel generators. Fuel.

[14] IARC Working Group on the Evaluation of Carcinogenic Risks to Humans (2012). Chemical agents and related occupations. IARC Monogr. Eval. Carcinog. Risks Hum..

[15] IARC Working Group on the Evaluation of Carcinogenic Risks to Humans (2014). Diesel and gasoline engine exhausts and some nitroarenes. IARC monographs on the evaluation of carcinogenic risks to humans. IARC Monogr. Eval. Carcinog. Risks Hum..

[16] Claxton L.D. (2015). The history, genotoxicity and carcinogenicity of carbon-based fuels and their emissions: Part 4—Alternative fuels. Mutat. Res. Rev. Mutat. Res..

[17] Schins R.P., Knaapen A.M. (2007). Genotoxicity of poorly soluble particles. Inhal. Toxicol..

[18] Topinka J., Milcova A., Schmuczerova J., Mazac M., Pechout M., Vojtisek-Lom M. (2012). Genotoxic potential of organic extracts from particle emissions of diesel and rapeseed oil powered engines. Toxicol. Lett..

[19] André V., Barraud C., Capron D., Preterre D., Keravec V., Vendeville C., Cazier F., Pottier D., Morin J.P., Sichel F. (2015). Comparative mutagenicity and genotoxicity of particles and aerosols emitted by the combustion of standard vs. Rapeseed methyl ester supplemented bio-diesel fuels: Impact of after treatment devices: Oxidation catalyst and particulate filter. Mutat. Res. Genet. Toxicol. Environ. Mutagen..

[20] Steiner S., Heeb N.V., Czerwinski J., Comte P., Mayer A., Petri-Fink A., Rothen-Rutishauser B. (2014). Test-methods on the test-bench: A comparison of complete exhaust and exhaust particle extracts for genotoxicity/mutagenicity assessment. Environ. Sci. Technol..

[21] Iba M.M., Caccavale R.J. (2013). Genotoxic bioactivation of constituents of a diesel exhaust particle extract by the human lung. Environ. Mol. Mutagen..

[22] Bao L., Xu A., Tong L., Chen S., Zhu L., Zhao Y., Zhao G., Jiang E., Wang J., Wu L. (2009). Activated toxicity of diesel particulate extract by ultraviolet a radiation in mammalian cells: Role of singlet oxygen. Environ. Health Perspect..

[23] Jalava P.I., Tapanainen M., Kuuspalo K., Markkanen A., Hakulinen P., Happo M.S., Pennanen A.S., Ihalainen M., Yli-Pirila P., Makkonen U. (2010). Toxicological effects of emission particles from fossil- and biodiesel-fueled diesel engine with and without doc/poc catalytic converter. Inhal. Toxicol..

[24] Kooter I.M., van Vugt M.A.T.M., Jedynska A.D., Tromp P.C., Houtzager M.M.G., Verbeek R.P., Kadijk G., Mulderij M., Krul C.A.M. (2011). Toxicological characterization of diesel engine emissions using biodiesel and a closed soot filter. Atmos. Environ..

[25] Palkova L., Vondracek J., Trilecova L., Ciganek M., Pencikova K., Neca J., Milcova A., Topinka J., Machala M. (2015). The aryl hydrocarbon receptor-mediated and genotoxic effects of fractionated extract of standard reference diesel exhaust particle material in pulmonary, liver and prostate cells. Toxicol. In Vitro.

[26] Lou H., Du S.Y., Ji Q., Stolz A. (2006). Induction of AKR1C2 by phase II inducers: Identification of a distal consensus antioxidant response element regulated by Nrf2. Mol. Pharmacol..

[27] Penning T.M. (2014). Human aldo-keto reductases and the metabolic activation of polycyclic aromatic hydrocarbons. Chem. Res. Toxicol..

[28] Longhin E., Capasso L., Battaglia C., Proverbio M.C., Cosentino C., Cifola I., Mangano E., Camatini M., Gualtieri M. (2016). Integrative transcriptomic and protein analysis of human bronchial BEAS-2B exposed to seasonal urban particulate matter. Environ. Pollut..

[29] Ma Q. (2013). Role of Nrf2 in oxidative stress and toxicity. Annu. Rev. Pharmacol. Toxicol..

[30] Li N., Wang M.Y., Oberley T.D., Sempf J.M., Nel A.E. (2002). Comparison of the pro-oxidative and proinflammatory effects of organic diesel exhaust particle chemicals in bronchial epithelial cells and macrophages. J. Immunol..

[31] Totlandsdal A.I., Lag M., Lilleaas E., Cassee F., Schwarze P. (2015). Differential proinflammatory responses induced by diesel exhaust particles with contrasting pah and metal content. Environ. Toxicol..

[32] Arrigo A.P. (2007). The cellular “networking” of mammalian hsp27 and its functions in the control of protein folding, redox state and apoptosis. Adv. Exp. Med. Biol..

[33] Krause K., Wasner M., Reinhard W., Haugwitz U., Dohna C.L.Z., Mossner J., Engeland K. (2000). The tumour suppressor protein p53 can repress transcription of cyclin B. Nucleic Acids Res..

[34] Karantanos T., Tanimoto R., Edamura K., Hirayama T., Yang G., Golstov A.A., Wang J.X., Kurosaka S., Park S., Thompson T.C. (2014). Systemic GLIPR1-ΔTM protein as a novel therapeutic approach for prostate cancer. Int. J. Cancer.

[35] Feng X., Liu X., Zhang W., Xiao W.H. (2011). P53 directly suppresses BNIP3 expression to protect against hypoxia-induced cell death. EMBO J..

[36] Galvagni F., Orlandini M., Oliviero S. (2013). Role of the AP-1 transcription factor FOSL1 in endothelial cell adhesion and migration. Cell Adhes. Migr..

[37] West M.D., Shay J.W., Wright W.E., Linskens M.H.K. (1996). Altered expression of plasminogen activator and plasminogen activator inhibitor during cellular senescence. Exp. Gerontol..

[38] Johannson K.A., Vittinghoff E., Lee K., Balmes J.R., Ji W., Kaplan G.G., Kim D.S., Collard H.R. (2014). Acute exacerbation of idiopathic pulmonary fibrosis associated with air pollution exposure. Eur. Respir. J..

[39] Hattori N., Mizuno S., Yoshida Y., Chin K., Mishima M., Sisson T.H., Simon R.H., Nakamura T., Miyake M. (2004). The plasminogen activation system reduces fibrosis in the lung by a hepatocyte growth factor-dependent mechanism. Am. J. Pathol..

[40] Cho H.Y., Reddy S.P., Yamamoto M., Kleeberger S.R. (2004). The transcription factor Nrf2 protects against pulmonary fibrosis. FASEB J..

[41] Lakatos H.F., Thatcher T.H., Kottmann R.M., Garcia T.M., Phipps R.P., Sime P.J. (2007). The role of ppars in lung fibrosis. PPAR Res..

[42] Devchand P.R., Ziouzenkova O., Plutzky J. (2004). Oxidative stress and peroxisome proliferator-activated receptors: Reversing the curse?. Circ. Res..

[43] Zhang L., Jin Y., Huang M., Penning T.M. (2012). The role of human Aldo-Keto reductases in the metabolic activation and detoxication of polycyclic aromatic hydrocarbons: Interconversion of pah catechols and pah o-quinones. Front. Pharmacol..

[44] Jiang H., Vudathala D.K., Blair I.A., Penning T.M. (2006). Competing roles of Aldo-Keto reductase 1A1 and cytochrome P4501B1 in benzo[a]pyrene-7,8-diol activation in human bronchoalveolar H358 cells: Role of akrs in p4501b1 induction. Chem. Res. Toxicol..

[45] Andrysik Z., Vondracek J., Marvanova S., Ciganek M., Neca J., Pencikova K., Mahadevan B., Topinka J., Baird W.M., Kozubik A. (2011). Activation of the aryl hydrocarbon receptor is the major toxic mode of action of an organic extract of a reference urban dust particulate matter mixture: The role of polycyclic aromatic hydrocarbons. Mutat. Res. Fund. Mol. M.

[46] Crane R., Gadea B., Littlepage L., Wu H., Ruderman J.V. (2004). Aurora A, meiosis and mitosis. Biol. Cell.

[47] Kunitoku N., Sasayama T., Marumoto T., Zhang D.W., Honda S., Kobayashi O., Hatakeyama K., Ushio Y., Saya H., Hirota T. (2003). Cenp-a phosphorylation by aurora-A in prophase is required for enrichment of aurora-b inner centromeres and for kinetochore function. Dev. Cell.

[48] Neef R., Preisinger C., Sutcliffe J., Kopajtich R., Nigg E.A., Mayer T.U., Barr F.A. (2003). Phosphorylation of mitotic kinesin-like protein 2 by polo-like kinase 1 is required for cytokinesis. J. Cell Biol..

[49] Wu C.C., Yang T.Y., Yu C.T., Phan L., Ivan C., Sood A.K., Hsu S.L., Lee M.H. (2012). P53 negatively regulates aurora a via both transcriptional and posttranslational regulation. Cell Cycle.

[50] Eyers P.A., Erikson E., Chen L.G., Maller J.L. (2003). A novel mechanism for activation of the protein kinase aurora A. Curr. Biol..

[51] Garrido G., Vernos I. (2016). Non-centrosomal tpx2-dependent regulation of the aurora a kinase: Functional implications for healthy and pathological cell division. Front. Oncol..

[52] Aguirre-Portoles C., Bird A.W., Hyman A., Canamero M., de Castro I.P., Malunnbres M. (2012). Tpx2 controls spindle integrity, genome stability, and tumor development. Cancer Res..

[53] Livak K.J., Schmittgen T.D. (2001). Analysis of relative gene expression data using real-time quantitative PCR and the 2^−ΔΔ*C*t^ method. Methods.

[54] Du P., Kibbe W.A., Lin S.M. (2008). Lumi: A pipeline for processing illumina microarray. Bioinformatics.

[55] Smyth G.K. (2004). Linear models and empirical bayes methods for assessing differential expression in microarray experiments. Stat. Appl. Genet. Mol. Biol..

[56] Chen J., Bardes E.E., Aronow B.J., Jegga A.G. (2009). Toppgene suite for gene list enrichment analysis and candidate gene prioritization. Nucleic Acids Res..

[57] R Core Team (2015). R: A Language and Environment for Statistical Computing.

[58] Chambers J.M., Freeny A., Heiberger R.M. (1992). Analysis of Variance.

[59] Benjamini Y., Hochberg Y. (1995). Controlling the false discovery rate: A practical and powerful approach to multiple testing. J. R. Stat. Soc..

[60] Yandell B.S. (1997). Practical Data Analysis for Designed Experiments.

