# Supplementary material for: Comparative Analysis of Toxic Responses of Organic Extracts from Diesel and Selected Alternative Fuels Engine Emissions in Human Lung BEAS-2B Cells
Source: Int J Mol Sci. 2016 Nov 3;17(11):1833. doi: 10.3390/ijms17111833 (PMC5133834; doi:10.3390/ijms17111833)
Supplement: Supplementary file 1 [file ijms-17-01833-s001.zip › ijms-147329-Supplementary Materials/ijms-147329-supp-final.pdf]

Supplementary Materials: Comparative Analysis of Toxic Responses of Organic Extracts from Diesel and Selected Alternative Fuels Engine Emissions in Human Lung BEAS-2B Cells

Helena Libalova, Pavel Rossner, Jr., Kristyna Vrbova, Tana Brzicova, Jitka Sikorova, Michal Vojtisek-Lom, Vit Beranek, Jiri Klema, Miroslav Ciganek, Jiri Neca, Katerina Pencikova, Miroslav Machala and Jan Topinka

(A)

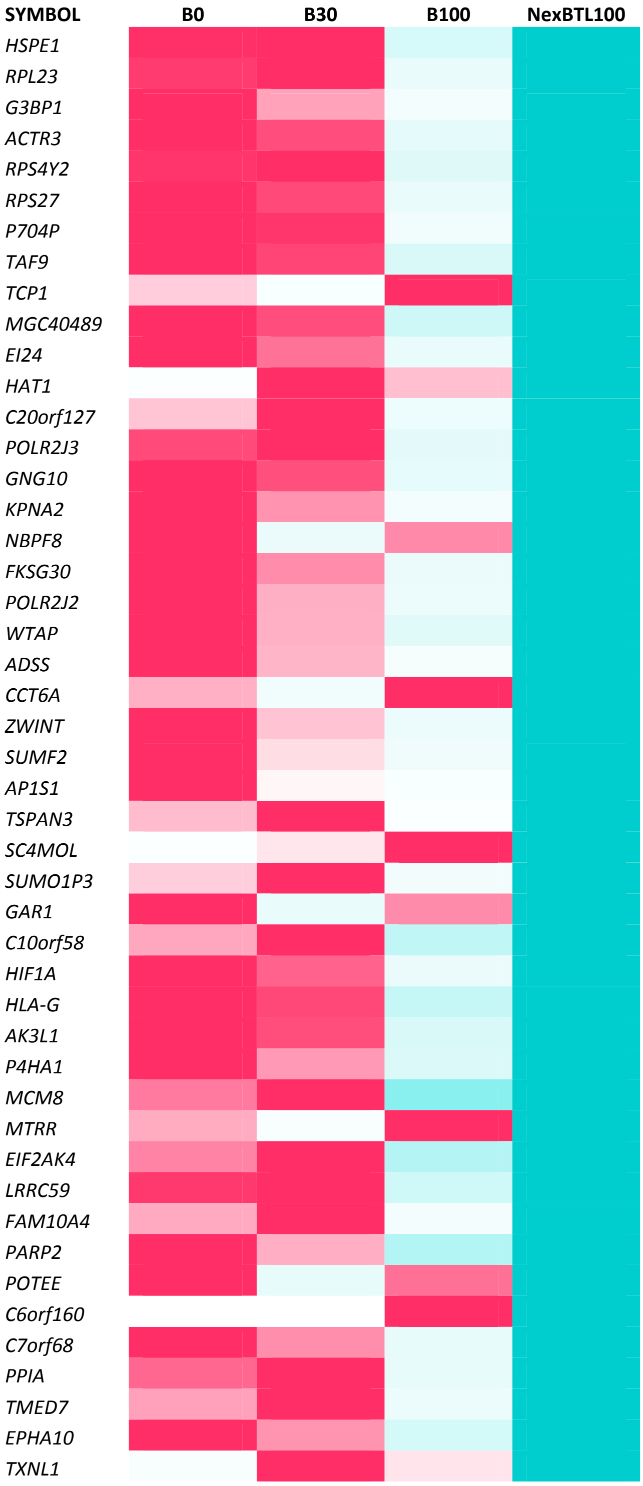

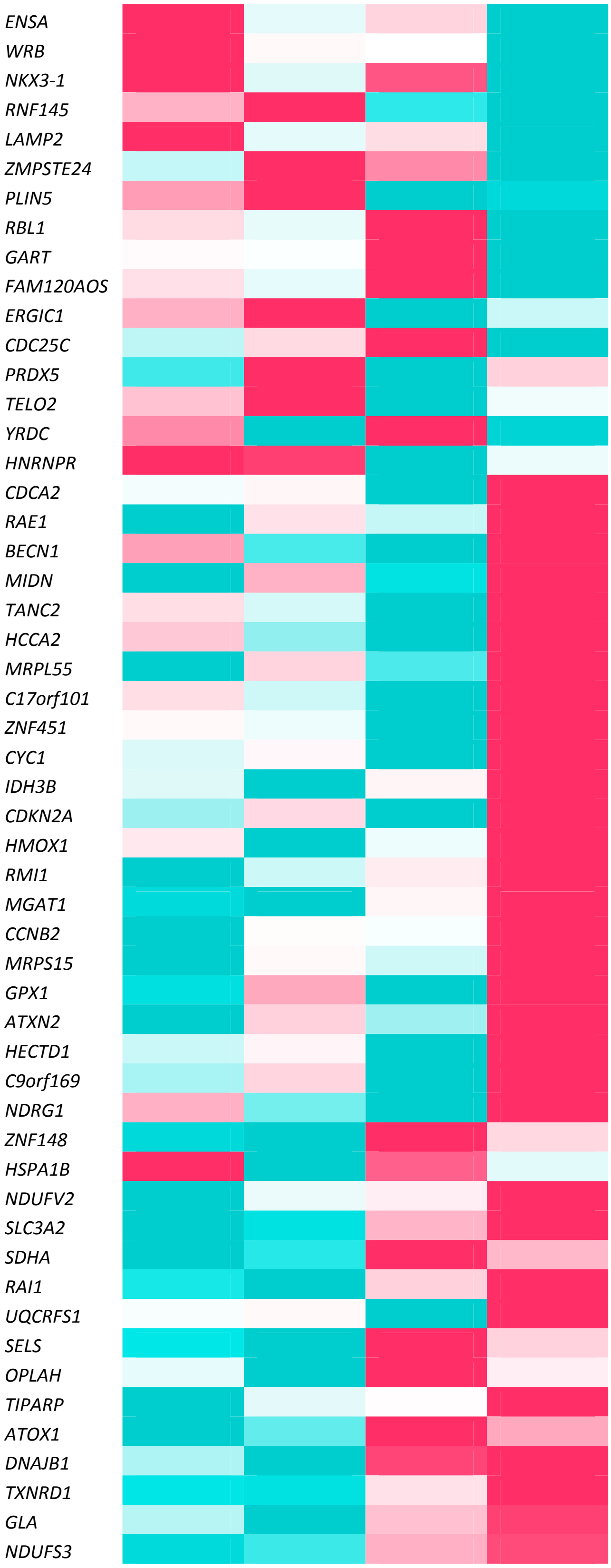
ENSA
WRB
NKX3-1
RNF145
LAMP2
ZMPSTE24
PLIN5
RBL1
GART
FAM120AOS
ERGIC1
CDC25C
PRDX5
TELO2
YRDC
HNRNPR
CDCA2
RAE1
BECN1
MIDN
TANC2
HCCA2
MRPL55
C17orf101
ZNF451
CYC1
IDH3B
CDKN2A
HMOX1
RMI1
MGAT1
CCNB2
MRPS15
GPX1
ATXN2
HECTD1
C9orf169
NDRG1
ZNF148
HSPA1B
NDUFV2
SLC3A2
SDHA
RAI1
UQCRFS1
SELS
OPLAH
TIPARP
ATOX1
DNAJB1
TXNRD1
GLA
NDUFS3

(**B**)

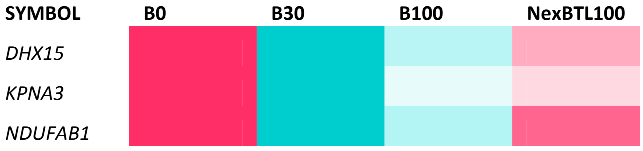
SYMBOL
B0
B30
B100
NexBTL100
DHX15
KPNA3
NDUFAB1

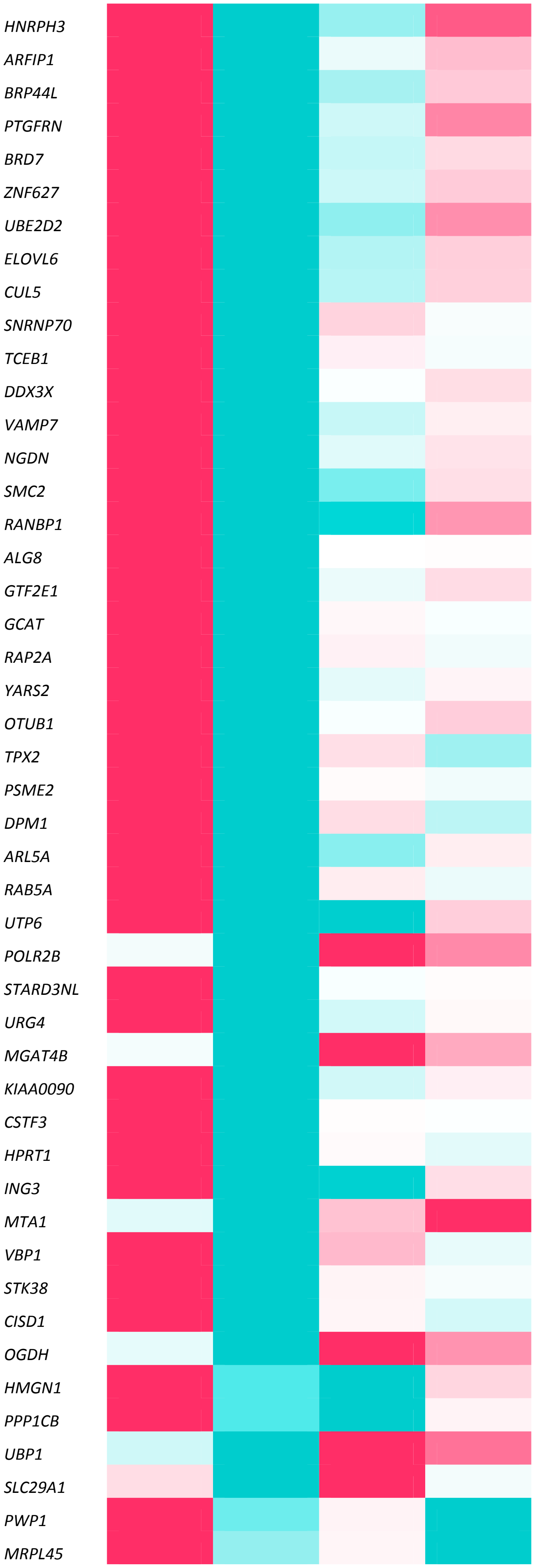
HNRPH3
ARFIP1
BRP44L
PTGFRN
BRD7
ZNF627
UBE2D2
ELOVL6
CUL5
SNRNP70
TCEB1
DDX3X
VAMP7
NGDN
SMC2
RANBP1
ALG8
GTF2E1
GCAT
RAP2A
YARS2
OTUB1
TPX2
PSME2
DPM1
ARL5A
RAB5A
UTP6
POLR2B
STARD3NL
URG4
MGAT4B
KIAA0090
CSTF3
HPRT1
ING3
MTA1
VBP1
STK38
CISD1
OGDH
HMGN1
PPP1CB
UBP1
SLC29A1
PWP1
MRPL45

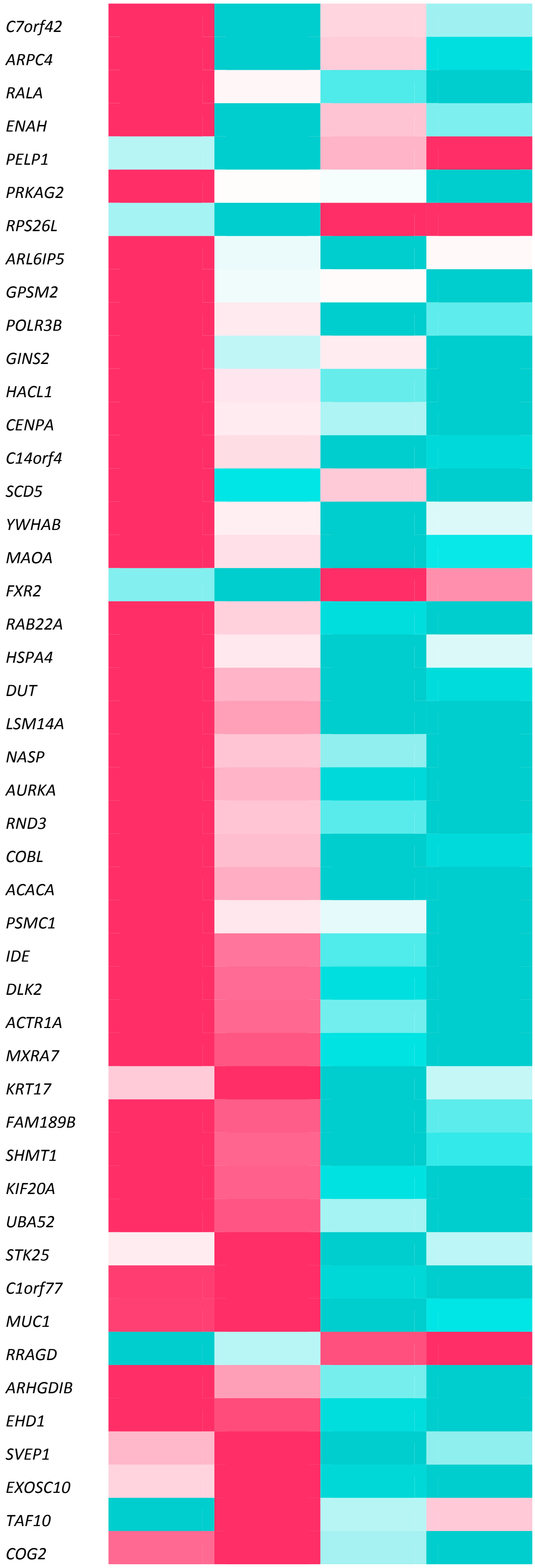
C7orf42
ARPC4
RALA
ENAH
PELP1
PRKAG2
RPS26L
ARL6IP5
GPSM2
POLR3B
GINS2
HACL1
CENPA
C14orf4
SCD5
YWHAB
MAOA
FXR2
RAB22A
HSPA4
DUT
LSM14A
NASP
AURKA
RND3
COBL
ACACA
PSMC1
IDE
DLK2
ACTR1A
MXRA7
KRT17
FAM189B
SHMT1
KIF20A
UBA52
STK25
C1orf77
MUC1
RRAGD
ARHGDIB
EHD1
SVEP1
EXOSC10
TAF10
COG2

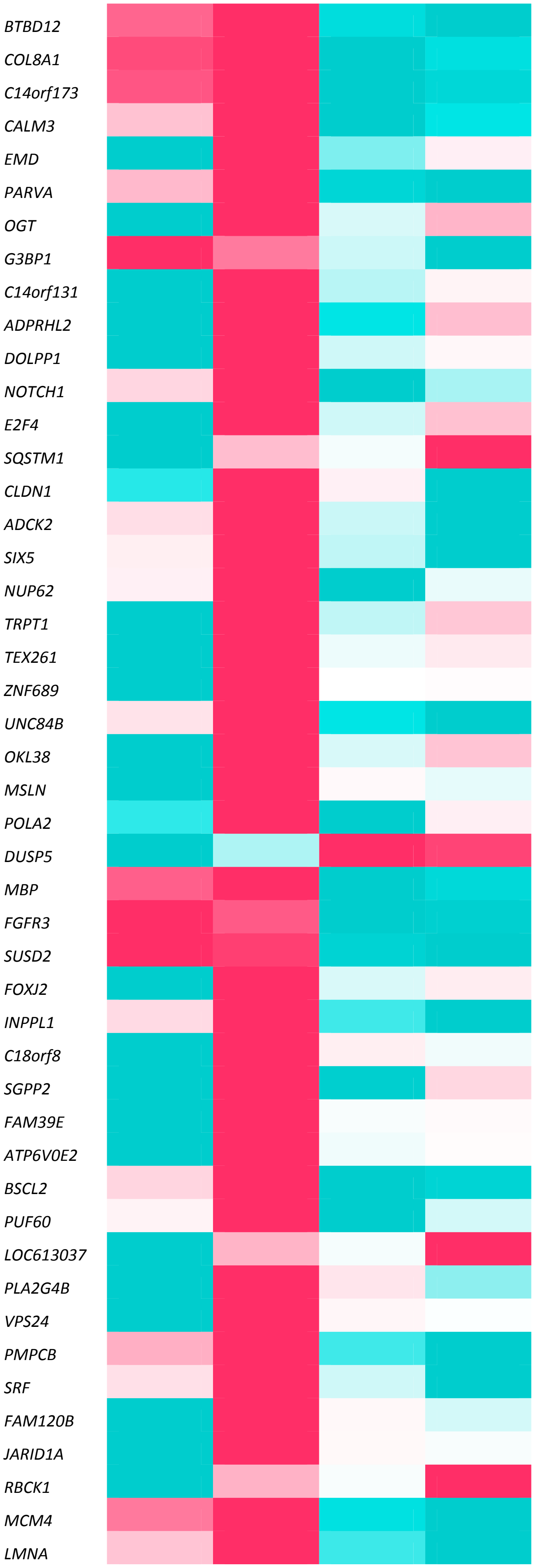
BTBD12
COL8A1
C14orf173
CALM3
EMD
PARVA
OGT
G3BP1
C14orf131
ADPRHL2
DOLPP1
NOTCH1
E2F4
SQSTM1
CLDN1
ADCK2
SIX5
NUP62
TRPT1
TEX261
ZNF689
UNC84B
OKL38
MSLN
POLA2
DUSP5
MBP
FGFR3
SUSD2
FOXJ2
INPPL1
C18orf8
SGPP2
FAM39E
ATP6V0E2
BSCL2
PUF60
LOC613037
PLA2G4B
VPS24
PMPCB
SRF
FAM120B
JARID1A
RBCK1
MCM4
LMNA

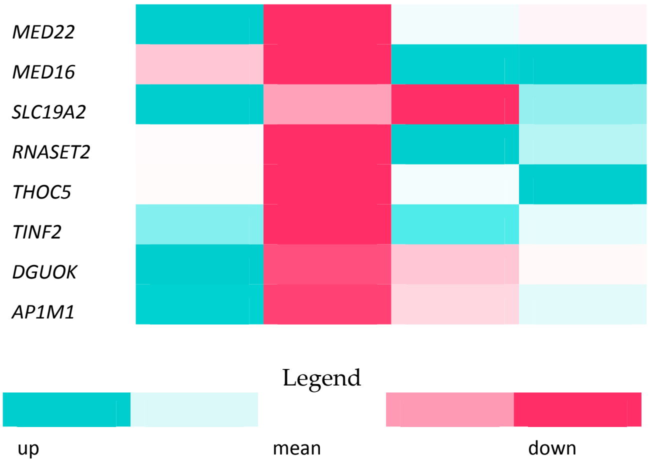

Figure S1. Top differentially expressed genes among diesel exhaust particles (DEP) extract treatments identified by one-way ANOVA (p value < 0.05, log FC > ± 1.5). Heat maps show relative expression of significantly modulated genes after (**A**) 4 h and (**B**) 24 h exposure to all DEP extracts. Each row corresponds to a gene (gene symbols are listed to the right of each row) and each column to a sample. The colors are scaled by row; red and blue indicate two standard deviations below or above the mean (white), respectively.

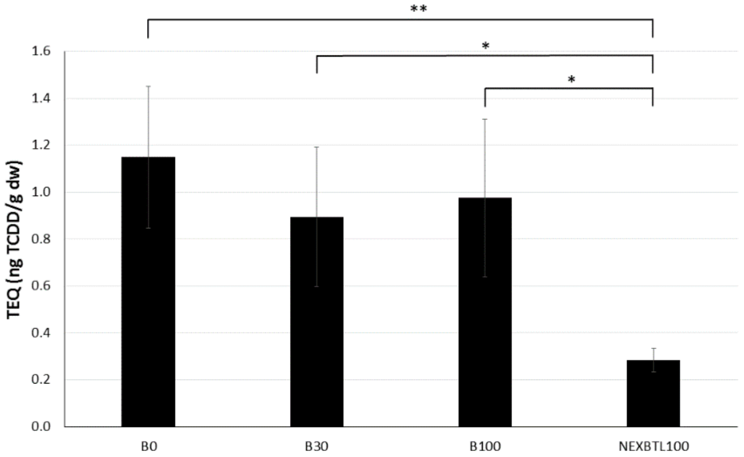

Figure S2. The aryl hydrocarbon receptor (AhR)-mediated activity of B0, B30, B100 and NEXTBTL100 extracts determined in human hepatoma HepG2 stable transfected by luciferase reporter gene under the control by the AhR (DR-CALUX assay). The activities were expressed as 2,3,7,8-tetrachlorodibenzo-p-dioxin (TCDD) equivalents (TEQs). * p-value < 0.05, ** p-value < 0.01.

Table S2. Sequences of primers used in RT-qPCR.

Symbol	RefSeq ID	Oligonucleotide	
AKR1C2	NM_001354.4	sense	GGTATTTATCAGTCAGTGCCTCTC
		antisense	TGCTAGTCAATATCGCTCATCCT
TXNRD1	NM_182743.1	sense	CTTGGATAGGAGTTGGTGAATAGAA
		antisense	GGGCTTGAGACTGGTGACTT
HMOX1	NM_002133.1	sense	GGAAGCCCCCACTCAACA
		antisense	GCATAAAGCCCTACAGCAACT
CYP1A1	NM_000499.2	sense	CAAGGTGTTAAGTGAGAAGGTG
		antisense	AGCAGGATAGCCAGGAAGAG
CYP1B1	NM_000104.2	sense	CACTGGAAACCGCACCTC
		antisense	AGCACCGACAGGAGTAGC
TPX2	NM_012112.4	sense	CAGGAGGAATATAAGGAAGTGAACT
		antisense	TGGGACAGGTTGAAAGGCTTA
CCNB2	NM_004701.2	sense	TGGAGAATATTGACACAGGAGTTAAT
		antisense	TGGGTTGAACTGGAACTTTGG
CDKN2A	NM_058195.2	sense	ATGTCCTGCCTTTTAACGTAGATA
		antisense	CTCACTCCAGAAAACTCCAACA
HSPE1	NM_002157.1	sense	ATTATGCTTCCAGAAAAATCTCAACG
		antisense	CTTTATCTCCAACTTTCACGCTAAC
FOSL1	NM_005438.2	sense	AGCCCAGAGACTTTGTAGATCCTT
		antisense	TTCTGTCAGGAGATAGGGTTGGG
BNIP3	NM_004052.2	sense	AGACCCCACAGGACACTAAC
		antisense	GACCTCTTTCCTCCTCTCCAT
